# Quantifying Microsecond Exchange in Large Protein Complexes with Accelerated Relaxation Dispersion Experiments in the Solid State

**DOI:** 10.1038/s41598-019-47507-8

**Published:** 2019-07-31

**Authors:** Carl Öster, Simone Kosol, Józef R. Lewandowski

**Affiliations:** Department of Chemistry, University of Warwick, Gibbet Hill Road, Coventry, CV4 7AL UK

**Keywords:** Solid-state NMR, Biophysical chemistry

## Abstract

Solid state NMR is a powerful method to obtain information on the structure and dynamics of protein complexes that, due to solubility and size limitations, cannot be achieved by other methods. Here, we present an approach that allows the quantification of microsecond conformational exchange in large protein complexes by using a paramagnetic agent to accelerate ^15^N *R*_1ρ_ relaxation dispersion measurements and overcome sensitivity limitations. The method is validated on crystalline GB1 and then applied to a >300 kDa precipitated complex of GB1 with full length human immunoglobulin G (IgG). The addition of a paramagnetic agent increased the signal to noise ratio per time unit by a factor of 5, which allowed full relaxation dispersion curves to be recorded on a sample containing less than 50 μg of labelled material in 5 and 10 days on 850 and 700 MHz spectrometers, respectively. We discover a similar exchange process across the β-sheet in GB1 in crystals and in complex with IgG. However, the slow motion observed for a number of residues in the α-helix of crystalline GB1 is not detected in the complex.

## Introduction

Protein dynamics play an important role in many biological processes such as enzymatic catalysis, ligand binding, or molecular recognition. Many motions implicated in these processes occur on microsecond or slower time scale and can be probed using chemical exchange based methods such as relaxation dispersion^1,2^. Applying these methods to slowly tumbling proteins and protein complexes above a few tens of kDa in solution becomes increasingly difficult due to the enhanced *T*_2_ relaxation resulting in size dependent broadening of NMR lines. In solid-state NMR, however, line broadening is independent of the size of the system. This allows the study of biomolecules in assemblies of several hundred kDa and beyond, provided the intrinsic challenges of sensitivity and resolution can be addressed successfully^3–11^. In solids, just as in solution, chemical exchange processes can be followed by observing the effects of modulation of isotropic chemical shift. In addition, they can be monitored through the effects of modulating anisotropic interactions, e.g. modulation of dipolar couplings in near-rotary resonance relaxation dispersion experiments (NERRD)^12,13^. The combination of the two approaches can yield a detailed view of microsecond motions^12^. Previously, we have shown that we can access the protein backbone dynamics of GB1 in a >300 kDa asymmetric complex with full length immunoglobulin G (IgG) by measuring ^15^N *R*_1_ and *R*_1ρ_ relaxation rates^3^. Proton detection at >50 kHz magic angle spinning (MAS) frequencies provided sufficient sensitivity enhancement to obtain data for a miniscule quantity of GB1 (~50 μg in a 1.3 mm rotor) in a reasonable timespan^4^. The strong spinning frequency dependence of ^15^N *R*_1ρ_^14^ suggested the presence of extensive slow μs motions of GB1 in the complex with IgG that are not present in GB1 crystals^3^. Since methods such as *R*_1ρ_ relaxation dispersion^15^ rely on recording numerous 2D (or 3D) spectra to measure *R*_1ρ_ relaxation rates at several different spin lock fields, they require unpractically long experimental times for large complexes (estimated to be on the order of one to two months of experimental time for our samples). This forced us to resort to more approximate, qualitative methods to detect the presence of microsecond exchange. However, the microsecond exchange detected in this way could not account for the observed spinning frequency dependence of *R*_1ρ_ and suggested the presence of an overall motion of GB1 in the complex^3^. To permit quantification of microsecond exchange through relaxation dispersion measurements on large protein complexes in more realistic time frames, we propose to add paramagnetic relaxation enhancement agents to the samples to accelerate ^15^N *R*_1ρ_ relaxation dispersion measurement. The addition of a paramagnetic agent such as copper ethylenediaminetetraacetate (CuEDTA) or gadolinium diethylenetriaminepentaacetic acid bismethylamide (Gd(DTPA-BMA)), shortens ^1^H *T*_1_’s to allow fast recycling of the experiments. This effect is frequently used in solid state NMR to speed up acquisition for chemical shift assignments and structure calculations^4,16–30^.

Since the paramagnetic agent also affects ^1^H and ^15^N *R*_2_’s (*R*_2_ = 1/*T*_2_), its concentration needs to be adjusted to avoid excessive line broadening (see Supplementary Fig. S1 for examples of line broadening after addition of Gd(DTPA-BMA)). Generally, due to the distance dependence of the paramagnetic effect, the presence of a paramagnetic agent is not desirable in experiments for quantifying site-specific dynamics from relaxation rates. However, the exchange contribution to *R*_2_ or *R*_1ρ_, which is the source of relaxation dispersion related to modulation of isotropic chemical shifts (or from anisotropic interactions in NERRD experiments)^2,15^, is not affected by the paramagnetic relaxation enhancement (provided it does not induce paramagnetic shifts). Thus, in principle, quantitative relaxation dispersion can still be measured in the presence of paramagnetic agents. The only difference between a relaxation dispersion experiment in the presence and absence of paramagnetic dopants should be a different base/plateau relaxation rate (*R*_1ρ,0_). While this manuscript was in preparation, a similar approach was used to characterize exchange in crystalline SH3^31,32^ but to the best of our knowledge, no extensive validation of it has been performed. Thus, to validate the proposed accelerated relaxation dispersion method, we first compared ^15^N *R*_1ρ_ relaxation dispersion experiments with and without paramagnetic agent on crystalline GB1 (in the following we refer to such samples as GB1_pre_ and GB1_dia_, respectively). Subsequently, we applied the validated technique to obtain site specific information on microsecond conformational exchange of GB1 in the >300 kDa complex with IgG.

## Results

We chose Gd(DTPA-BMA) as paramagnetic dopant over the, in solid state NMR, more popular CuEDTA because Gd(DTPA-BMA) is a more inert probe. The overall negatively charged CuEDTA has in some instances been reported to bind specifically to proteins^33,34^ or even disrupt assembly of certain systems (T. Polenova – private communication). The absence of any chemical shift changes in either crystalline GB1 or precipitated GB1:IgG complex upon addition of Gd(DTPA-BMA) indicates that the dopant does not interact specifically with our samples (see also Supplementary Fig. S1).

To determine the optimal concentration of the dopant, we have examined a number of samples with varying concentrations of Gd(DTPA-BMA). The optimal concentration of paramagnetic compound that results in the best compromise between shortening of ^1^H *T*_1_ and resolution was determined experimentally for each system. The overall broadening seems to be dependent on the specific assemblies (or rather the specific pattern of solvent accessibility). For example, 5 mM Gd(DTPA-BMA) induced levels of broadening acceptable for site specific analysis in the GB1:IgG complex but caused too much broadening in crystalline GB1 (see Supplementary Fig. S1). Paramagnetic doping may also affect the efficiency of the polarization transfer steps and lead to loss of signal. Consequently, we have also compared the overall sensitivity per unit time for each sample. Higher concentrations (2–5 mM) of Gd(DTPA-BMA) gave similar signal to noise ratios (SNRs) per unit time for GB1:IgG corresponding to an increase of approximately 5 times compared to a sample without Gd(DTPA-BMA) (see Supplementary Table S1 for a comparison of SNRs). Similar concentrations of the dopant resulted in severe line broadening for crystalline GB1, suggesting that the optimal concentration for crystalline GB1 samples is most likely less than 2 mM.

**Figure 1:**
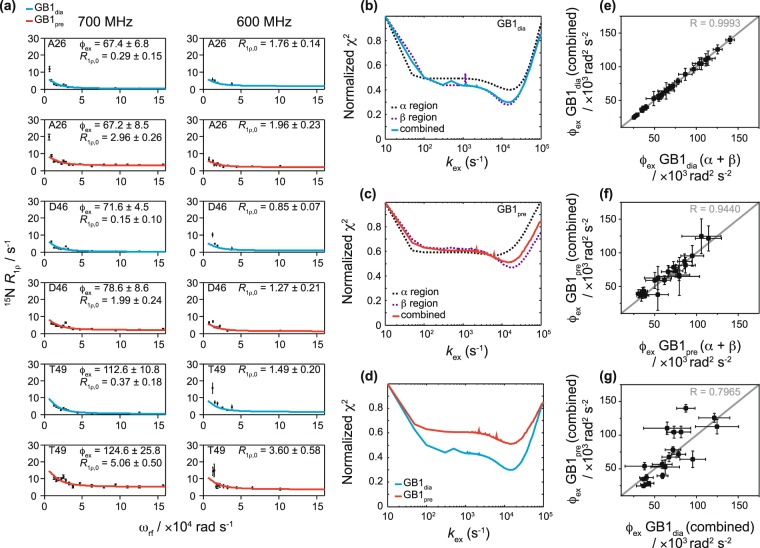
^15^N *R*_1ρ_ relaxation dispersion for crystalline GB1. (**a**) Example fits based on the data from the sample without paramagnetic dopant (GB1_dia_) (blue lines, *k*_ex_ = 14 ± 0.5 × 10^3^ s^−1^) and the sample with paramagnetic dopant (GB1_pre_) (red lines, *k*_ex_ = 15 ± 2 × 10^3^ s^−1^) obtained from measurements at 600 MHz (14.1 T) and 700 MHz (16.4 T) and a sample temperature of 300 ± 2 K. For simplicity the units for the residue specific *φ*_ex_ and *R*_1ρ,0_ are omitted in the graphs and are ×10^3^ rad^2^ s^−2^ and s^−1^, respectively. (**b**–**d**) Agreement between experimental and fitted data represented by plots between fixed exchange coefficients, *k*_ex_, and the corresponding normalized χ^2^ values from joint fitting of relaxation dispersion curves for different groupings of residues in GB1_dia_ (**b**) and GB1_pre_ (**c**), as well as a comparison of normalised χ^2^ values from global fits in GB1_dia_ and GB1_pre_ (**d**). The fits obtained from the different groupings are indicated with dotted black lines for the α-helix region, dotted purple lines for the β–sheet region and solid lines for all residues together (blue for GB1_dia_ and red for GB1_pre_). (**e**, **f**) Correlation plots for *φ*_ex_ values based on fits of all residues together and the α-helix and β-sheet regions separately for GB1_dia_ (**e**) and GB1_pre_ (**f**). (**g**) Correlation plot for *φ*_ex_ values based on fits of all residues together between GB1_dia_ and GB1_pre_. The correlation coefficient (R) is indicated in grey in each correlation plot.

Relaxation dispersion measurements in the solid state can contain contributions from both, incoherent effects originating from dynamic processes and coherent effects originating from dipolar couplings that are not completely averaged by magic angle spinning^35^. Consequently, in order to obtain quantitative relaxation dispersion measurements, the experiments need to be performed under conditions where coherent effects are suppressed^35,36^. For ^15^N measurements, this is typically achieved by deuteration of the studied protein and partial re-protonation at exchangeable sites and application of fast, >40 kHz, spinning^15^. For larger concentrations of remaining protons in the sample, faster spinning frequencies need to be employed. Here, we apply 60 kHz MAS to 100% back-exchanged perdeuterated [U-^13^C,^15^N]GB1 samples. As can be seen by the flat dispersion curves for residues not undergoing conformational exchange (see Supplementary Figs S2–7 for plots of *R*_1ρ_ as a function of spin-lock frequencies for all samples), the dipolar dephasing is sufficiently suppressed under these conditions. Generally, residues that show clear relaxation dispersion in experiments with Gd(DTPA-BMA), exhibit the same behavior in control experiments without paramagnetic dopant (see Supplementary Fig. S8; note that some residues had to be excluded from the analysis if their peaks were heavily overlapped). We observed dispersion in two regions in crystalline GB1: the α-helix (α-helix region, residues 24, 26, 28, 29, 32, 33, 35, 36 in GB1_pre_) and strands 2–4 of the β-sheet and the loops connecting them (β-sheet region; residues 12, 17–18, 44–46, 49–53 in GB1_pre_). For some residues, raised rates were observed near spin locks ~5 kHz (3.14 × 10^4^ rad s^−1^), e.g. in D46 (Fig. 1a). Similar features were observed in the same regions for different samples of crystalline GB1, measured at different conditions, suggesting that they may not be due to random error. At the same time, in spite of extensive control measurements, we were not able to identify the origin of such features or reproduce them in simulations.

To quantify the microsecond exchange processes in crystalline GB1 in diamagnetic and paramagnetic samples, we fitted the data for residues showing clear dispersion at two magnetic fields (14.1 and 16.4 T) to the two-site exchange Bloch-McConnell formalism (eq. 1, see Methods)^37,38^. All residues were fitted individually and in groups. Residues that were close in space displayed very similar exchange rates when fitted individually (see Supplementary Table S2) and were consequently clustered into groups to fit them simultaneously. This is permissible, assuming that the clustered residues undergo a common motion, or, more specifically, share a common exchange rate, *k*_ex_, and have the same populations of exchanging states, *p*_A_ & *p*_B_, but with residue specific chemical shift differences between the states, Δ*δ*. Because the populations and chemical shift differences are highly correlated and difficult to extract from the fits, *φ*_ex_, the product between *p*_A_, *p*_B_ and Δ*δ*^2^ is used as a single fit parameter. We fitted residues in the α-helix and β-sheet regions separately and together (see Fig. 1a for representative relaxation dispersion curves for GB1_dia_ and GB1_pre_ and Supplementary Tables S2–9 and Figs S9–10 for results from all residues). The obtained *k*_ex_ for GB1_dia_ were very similar between the different group fits: 14 ± 0.5 × 10^3^ s^−1^ for all residues together, 14 ± 1 × 10^3^ s^−1^ for the β-sheet region and 16 ± 1 × 10^3^ s^−1^ for the α-helix region. For GB1_pre_ there were, however, some differences between the group fits: 15 ± 2 × 10^3^ s^−1^ for all residues together, 14 ± 2 × 10^3^ for the β-sheet region and 6 ± 2 × 10^3^ s^−1^ for the α-helix region. To investigate the difference in *k*_ex_ rates between the α-helix and β-sheet regions in GB1_pre_, we plotted the χ^2^ values (see Eq. 8 in the Methods part) as a function of varied *k*_ex_ (Fig. 1b, c). For GB1_dia_ the lowest χ^2^ values (equivalent to the best fits) are obtained for the same *k*_ex_ values regardless of whether the α helix and β sheet regions are fitted individually or together. However, if the α region of GB1_pre_ is fitted alone (Fig. 1c, dotted black line), the curve has a shallow minimum suggesting *k*_ex_ is not well restrained by the data in this case. Correlation plots between the obtained *φ*_ex_ values from fits of the α helix and β sheet regions separately and all residues together show excellent agreement with correlation coefficients (R values) of 0.9993 and 0.9440 for GB1_dia_ (Fig. 1e) and GB1_pre_ (Fig. 1f), respectively. To further evaluate the different models, we calculated Bayesian Information Criterion values (BIC, see Methods, Eq. 9). The lowest BIC values were yielded by a global group fit including all residues showing dispersion (see Supplementary Table S10), suggesting that, statistically, this is the best model. A comparison between the global fits for all residues exhibiting dispersion in GB1_dia_ and GB1_pre_ (Fig. 1d) show clear narrow minima in the same regions of *k*_ex_. Overall, the parameters of the exchange processes obtained from GB1_pre_ agree reasonably well with the data for GB1_dia_ with excellent agreements between the *k*_ex_ values (14 ± 0.5 × 10^3^ s^−1^ for GB1_dia_ and 15 ± 2 × 10^3^ s^−1^ for GB1_pre_) and a good correlation between *φ*_ex_ values for most residues (Fig. 1g). This confirms that although the paramagnetic agent affects *R*_1ρ_ relaxation rates, it does not affect the exchange contribution.

Encouraged by the reasonable agreement for the microsecond exchange between crystalline GB1_dia_ and GB1_pre_, we applied the accelerated relaxation dispersion approach to a more challenging system, GB1 in a >300 kDa complex with IgG. Performing ^15^N *R*_1ρ_ relaxation dispersion on a diamagnetic sample of the complex would require unpractically long experimental times with each *R*_1ρ_ measurement requiring ~3–5 days to obtain SNRs sufficient for quantitative analysis^3,39^. Experimental times on the order of 1–2 months would be necessary for full relaxation dispersion measurement. Even if such long times could be dedicated to a single experiment, maintaining sufficient experimental stability over the duration is very challenging. The addition of Gd(DTPA-BMA) yielded an increase in SNR per time unit of about 5 in the complex (based on 2D HN correlation spectra, see Supplementary Table S1). This allowed us to perform these experiments in 5 days on an 850 MHz spectrometer (20 T) on a sample with 5 mM Gd(DTPA-BMA) and in 10 days on a 700 MHz spectrometer (16.4 T) on a sample with 2 mM Gd(DTPA-BMA), using 10 different spin-lock field strengths at each *B*_0_ field (experimental durations are shown in Supplementary Table S10 and examples of *R*_1ρ_ fits are shown in Supplementary Fig. S.11).

In our experiments, many of the residues that exhibit relaxation dispersion in GB1 crystals also do so in the complex with IgG. In the β-sheet region, dispersion was observed for the same residues with the exception of residue 38, which is located in the loop region between the α-helix and the β3 strand. Gly38 showed clear dispersion in the GB1:IgG complex but not in crystalline GB1 (see Supplementary Figs S2–9; note that data for some residues that show μs exchange in crystalline GB1 could not be obtained due to either severe overlap or insufficient signal to noise in the GB1:IgG complex, including several residues in the α helix). In addition, the dispersion at the C-terminal end of β4 in the complex was less clear compared to crystalline GB1, especially at the lower magnetic field. A possible explanation for this discrepancy could be the presence of additional intermolecular interactions in the complex that either slow down conformational exchange or restrict its amplitude. We have recently proposed the existence of such an additional interaction interface in the complex with full length IgG, based on chemical shift perturbations^4^ and solvent PREs^39^. Interestingly, in contrast to crystalline GB1, in the complex none of the residues in the α-helix showed clear dispersion at either *B*_0_ field (see Supplementary Fig. S12 for a summary of which residues show dispersion), indicating that the motion we observed for the α helix in the crystalline sample is either too slow to detect in experiments with the lowest spin lock frequency of ~2 kHz or is not present at all in the complex.

**Figure 2:**
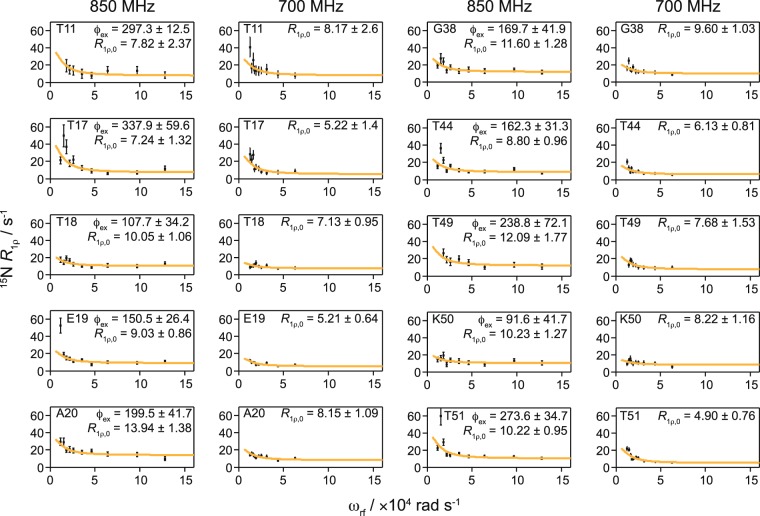
^15^N *R*_1ρ_ relaxation dispersion profiles for GB1 in complex with IgG. Experiments were acquired at 700 MHz (16.4 T) with 2 mM Gd(DTPA-BMA) and 850 MHz (20 T) with 5 mM Gd(DTPA-BMA), and a sample temperature of 300 ± 2 K. Best fit curves to a two-site exchange model assuming a common motion are shown as orange lines (*k*_ex_ = 13 ± 3 × 10^3^ s^−1^). Residue specific *φ*_ex_ (×10^3^ rad^2^ s^−2^) and *R*_1ρ,0_ (s^−1^) are indicated in each plot.

Since all residues showing dispersion in the GB1:IgG complex were located in the β-sheet region, we considered them as being involved in the same motion and fitted them together to obtain a single value for *k*_ex_ (13 ± 3 × 10^3^ s^−1^; see Fig. 2). The best fit parameters for GB1:IgG are listed in Supplementary Tables S12,13 (the fits for individual residues are also included for completeness). To investigate if there were separate motions of the individual binding sites to the Fab and Fc fragments of IgG, we grouped the residues close to each binding site and fitted each group separately. This yielded essentially identical results to fits of all residues combined (see Supplementary Tables S14,15 and Fig. S13).

**Figure 3:**
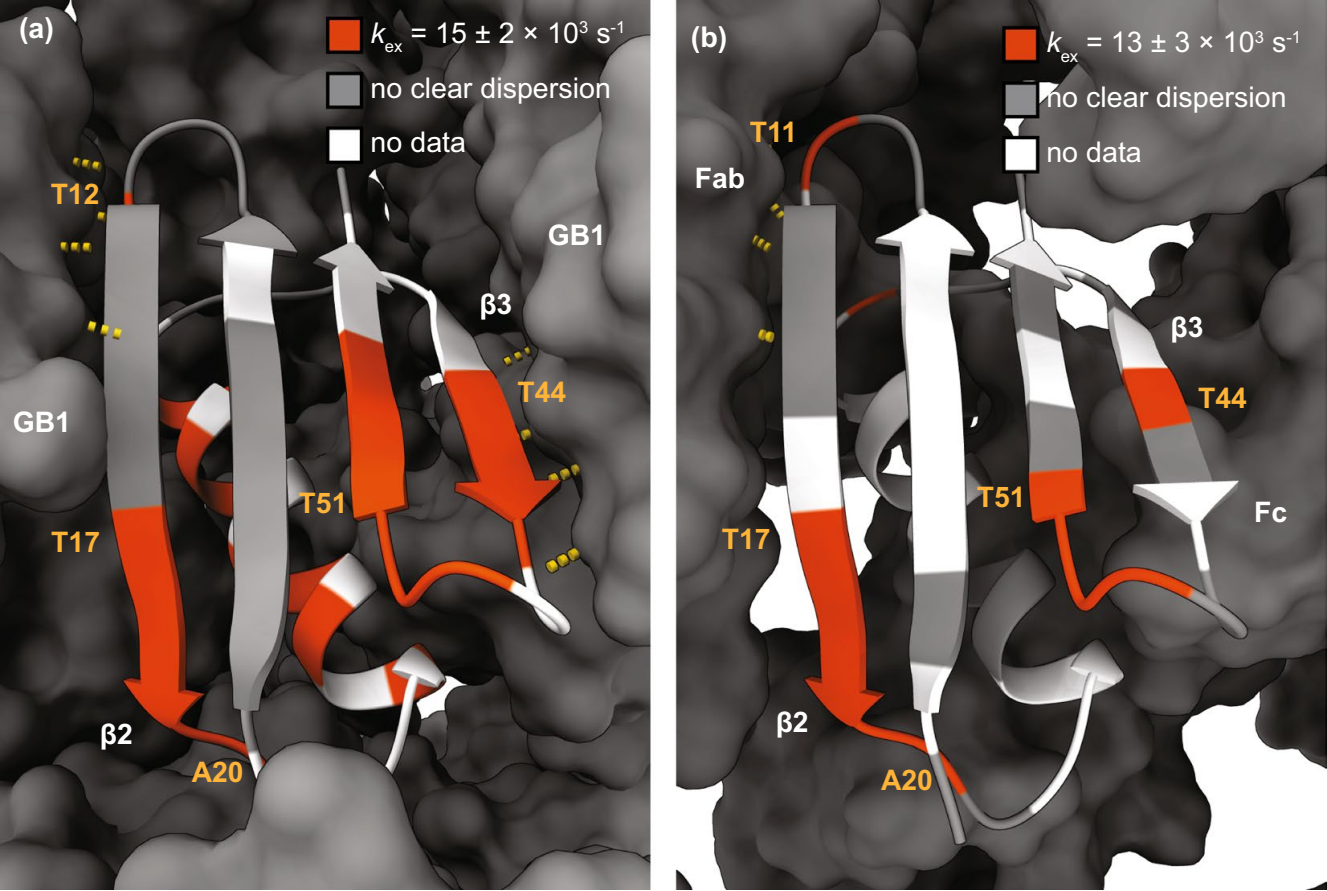
Microsecond exchange in crystalline GB1 (**a**) and GB1 in a complex with IgG (**b**). Residues showing relaxation dispersion are shown in orange. Exchange rates for group fits of the regions are given above the figures. White indicates residues for which data are missing. Grey represents residues with no clear relaxation dispersion. Yellow dashed lines represent hydrogen bonds.

Overall, we observed relaxation dispersion in similar regions as in earlier, qualitative measurements of GB1 in complex with IgG^3^. However, several false positives from this approximate method, together with need of quantification, highlight the importance of measuring full relaxation dispersion curves.

## Discussion

Somewhat unexpectedly, the results of ^15^N *R*_1ρ_ relaxation dispersion experiments suggest that, in crystalline GB1 and in GB1 in the complex with IgG, slow conformational exchange occurs for nearly identical sets of residues in the β-sheet. Moreover, the process is characterized by similar microsecond exchange rates. A direct comparison of these results to an isolated molecule of GB1 in solution is not possible because, to the best of our knowledge, relaxation dispersion for the same GB1 construct has not been reported in the literature. The most closely related protein for which such solution NMR data are available is GB3 that has the same fold as GB1 but differs by 6 residues^40^. Such differences could potentially lead to modification of dynamic behavior of the protein but comparison of patterns of motions could still be informative. No microsecond exchange was detected at room temperature for GB3^40^. Low microsecond exchange was detected in loop 1 of GB3 only under supercooled conditions (residues 9–13, which are the same between GB1 and GB3)^40^. Extrapolated to room temperature, this exchange process would occur on the order of hundreds of nanoseconds, which is too fast to induce relaxation dispersion in the here considered regime. Indeed, in the crystalline sample of GB1, we do not observe relaxation dispersion for any residues in loop 1, with the exception of Leu12. This is consistent with extended model free analysis of ^15^N and ^13^C’ relaxation rates in crystalline GB1 at room temperature, which places the slow backbone motions in loop 1 in the range of hundreds of nanoseconds^41^. Thus, the available solution NMR relaxation dispersion data do not help to shed light on the potential origins of the microsecond motions detected in the solid state. The most likely explanation for the motions observed in the solid-state is that intermolecular interactions lead to increased energy barriers between subsets of interconverting conformations. Higher energy barriers would cause the concerned motions to be slower than in an isolated molecule in solution. The influence of intermolecular interactions on slow motions has been observed previously: in ubiquitin, the main slow conformational exchange process is present in solution and in crystals but appears much slower in the latter^13,15,42^. In the case of GB1, different intermolecular interactions seem to lead to a slowing down of a subset of motions that are too fast to be picked up by relaxation dispersion in solution. A combination of RDC measurements and molecular dynamics simulations of GB3 and GB1 in solution has suggested a presence of slow correlated motions across the β-sheet^43–45^. These motions are the most likely to be slowed down by intermolecular interactions to yield patterns observable through relaxation dispersion in the solid state. The characteristics of the motions in the β-sheet region in crystalline GB1 and the GB1:IgG complex are the most similar near the β2 strand. There, the pattern of intermolecular hydrogen bonds between two GB1 molecules in the crystal is very similar to the hydrogen bonds present between GB1 and the Fab fragment of IgG in the complex. The differences in the pattern of microsecond motions increase towards the β3 strand, where a set of intermolecular hydrogen bonds is present between GB1 molecules in the crystal but a different interaction interface is formed with the Fc fragment in the complex. The microsecond exchange observed for residues in the α-helix in crystalline GB1 and the apparent absence of similar motions in the complex could also be linked to different intermolecular interactions. In the crystals, the helix is packed more densely than in the complex with IgG^4,39,46,47^, which likely leads to increased energy barriers for helix motions in the crystal. Finally, we observe differences between *φ*_ex_ values in crystalline GB1 and GB1 in complex with IgG. These parameters are linked to the populations of the exchanging states and the chemical shift difference between the states. In crystalline GB1 the *φ*_ex_ values are between 25–140 × 10^3^ rad^2^ s^−2^, with the lower values in the α-helix and higher values in β strands and loop regions. The *φ*_ex_ values for GB1 in complex with IgG are significantly larger, 90–300 × 10^3^ rad^2^ s^−2^, suggesting that either the population of the minor state or the chemical shift differences between the states (or both) are larger in the complex than in the crystals.

In general, the presence of μs motions should induce spinning frequency dependence of *R*_1ρ_^14^. Whereas previously we observed a very strong spinning frequency dependence of *R*_1ρ_ rates in the GB1:IgG complex, most residues in crystalline GB1 showed, within the experimental error, weak or no spinning frequency dependence (see also Supplementary Fig. S16)^3^. In order to see how we can reconcile these data, we modelled ^15^N *R*_1ρ_ rates for residues showing dispersion in the β-sheet in crystalline GB1 using the simple model free formalism. Fitting of the data for each residue (at 39, 50 and 56 kHz MAS speeds at 14.1 T and 50 kHz and 60 kHz at 16.4 T, sample temperature ~300 K, data from ref.^3^) with the time scale fixed to the inverse of the average of *k*_ex_ for GB1_dia_ and GB1_pre_ (1/(15 × 10^3^ s^−1^) = 67 μs), the best-fit order parameters for the slow motions are between 0.998–0.983. To obtain such high order parameters, either the amplitude of the motion needs to be very small when the population of the minor state is relatively large or the amplitude of motion can be larger when the population of the minor state is small^48^. For example, in a two-site jump model with unequal populations, those order parameters correspond to a jump angle between 7–20° for a minor population of 5% and a jump angle of 3–9° for equal populations of the two states (see Supplementary Fig. S14).

The ^15^N *R*_1ρ_ relaxation dispersion data suggest that in both samples, crystalline GB1 and GB1 in the complex with IgG, microsecond motions are present and span the β-sheet at 300 K. Whereas at 300 K the data do not suggest additional microsecond motions in crystalline GB1 beyond the ones detected through relaxation dispersion, the spinning frequency dependency of ^15^N *R*_1ρ_ for GB1 in the complex is consistent with the presence of an additional small amplitude overall motion in the microsecond range. For many sites such an overall motion would not induce significant isotropic chemical shift modulation (since no local conformation change is involved) and thus would not lead to relaxation dispersion in the regime considered here^3^. On a side note, the bulk ^15^N NERRD data obtained on crystalline GB1 at a lower spinning frequency (20 kHz) and lower sample temperature (293 K) was previously attributed to ultralow amplitude overall rocking of the molecule^49^. However, under conditions employed in ref.^3^ (40–60 kHz MAS and 300 K) such a motion would result in negligible spinning frequency dependence of ^15^N *R*_1ρ_ (see also Supplementary Fig. S16 where these data are replotted).

In summary, we have shown that the addition of a paramagnetic agent to hydrated solid state NMR samples permits relaxation dispersion measurements in systems such as large protein complexes, where otherwise low sensitivity prevents data collection in a realistic time frame. In our sample, a >300 kDa precipitated complex of GB1:IgG, the increase in SNR per time unit was on the order of 5, which allowed us to obtain quantitative measurements in 1–2 weeks on samples containing a few dozens of micrograms of labelled GB1 in the complex. Our experiments revealed the presence of similar microsecond motions in the β-sheet in both crystalline GB1 and in the GB1:IgG complex. In contrast, microsecond motions in the α-helix observed in crystalline GB1 appear to be absent in GB1 in the complex with IgG. Overall, the microsecond motions detected using relaxation dispersion do not account for the large differences in ^15^N *R*_1ρ_ measured in the two different assemblies^3^, supporting the idea of an additional small amplitude overall motion being present in the GB1:IgG complex. Overall, these results highlight the importance of direct measurements in complex assemblies to complement studies of their isolated components. Such experiments aid our understanding of the dynamics in interacting systems on a molecular level. Our approach will enable similar measurements on other complex systems, which are beyond the reach of current approaches.

## Methods

### Sample preparation

Isotope labelled [U-^2^H,^13^C,^15^N]GB1 2Q6I was expressed as described before^39^. In brief, BL21(DE3) cells transformed with pGEV2^50^ were grown in LB until they reached an OD600 > 1.0. The cells were washed once with PBS before resuspension in M9 with D_2_O, deuterated [U-^13^C]glucose and ^15^NH_4_Cl and expression was induced with 0.5 mM IPTG after 1 h incubation at 37 °C. The protein was expressed for 4 h at 37 °C before the cells were harvested by centrifugation (4000 × G for 20 min at 16 °C) and lysed by sonication (50 mM potassium phosphate; 200 mM NaCl; 1 mg/ml lysozyme; pH 7.0). After heat treatment at 75 °C for 10 min, the lysate was cleared by centrifugation (12000 × G for 50 min). GB1 was precipitated over night with 80% ammonium sulfate and collected by centrifugation (15000 × G for 50 min). The pellet was resuspended in buffer (50 mM potassium phosphate; 200 mM NaCl; pH 7.0), and purified on a 16/600 Sephadex pg75 (GE Healthcare) gel filtration column. Fractions containing GB1 were collected, desalted, freeze-dried and stored at −20 °C.

Freeze-dried [U-^2^H,^13^C,^15^N]GB1 was dissolved in buffer (50 mM sodium phosphate buffer; pH 5.5) to obtain a protein concentration of 10 mg/ml and crystallized with the aid of 2:1 2-methyl-2,4-pentanediol (MPD):Isopropanol^51^. The GB1:IgG complex was formed by mixing GB1 and IgG (Sigma – Aldrich, lyophilized, human serum) solutions in a 2:1 molar ratio^4^. Crystalline GB1 and precipitated GB1:IgG complex were packed into NMR rotors using the following procedure: The crystals/precipitate were collected by centrifugation (1 min at 20 000 × G using a bench top centrifuge), and resuspended in a small volume of the supernatant containing 2% DSS and Gd(DTPA–BMA) at the desired concentration. The samples were transferred into 200 μl pipette tips, which were then attached to the rotors, put into 1.5 ml Eppendorf tubes and centrifuged (20 000 × G) in 1–4 minutes intervals until the rotors were full. The rotor caps were sealed with a silicone-based glue to prevent leakage.

### Solid state NMR

Solid state NMR spectra were recorded at 600 MHz Bruker Avance II+, 700 MHz Bruker Avance III HD and 850 MHz Bruker Avance III spectrometers, using Bruker 1.3 mm triple resonance probes at 60 kHz magic angle spinning. A Bruker BCU-X cooling unit was used to regulate the internal sample temperature to 27 ± 2 °C (measured from the chemical shift of water with respect to DSS). For experiments recorded at 700 MHz ^1^H Larmor frequency with the GB1:IgG complex, 10% D_2_O was added to the sample buffer before packing the rotors and deuterium locking was used in the same way as in solution NMR. ^15^N-^1^H 2D correlation spectra were recorded using a proton-detected heteronuclear correlation sequence. Double quantum cross-polarization (CP) contact times were between 0.5–1.5 ms and individually optimized for each sample. Recycle delays between 0.2–2 s were used depending on the amount of paramagnetic agent and magnetic field (see Supplementary Table S11). The optimal recycle delay for each sample was selected by comparing signal to noise ratios in 1D planes recorded with the same sequence as the 2D ^1^H-^15^N correlation experiments. *R*_1ρ_ relaxation dispersion experiments were recorded as several pseudo 3Ds with the varying spin-lock lengths as the 3^rd^ dimension and with different spin-lock power in each 3D experiment or a pseudo 4D (GB1:IgG complex at 850 MHz ^1^H Larmor frequency) with the varying spin-lock power as the 4^th^ dimension. In all solid-state experiments, hard pulses were applied at nutation frequencies of 100 kHz for ^1^H and 83.3 kHz for ^15^N. 10 kHz WALTZ-16 decoupling was applied on protons during ^15^N evolution, and on the ^15^N channel during direct ^1^H acquisition, while quadrature detection was achieved using the States-TPPI method. Suppression of the water signal was achieved by saturation with 50–200 ms of slpTPPM ^1^H decoupling applied at an amplitude of ¼ of the MAS frequency^52^ or 100–140 ms MISSISSIPPI^53^ at an amplitude of ½ the MAS frequency on resonance with the water signal. *R*_1ρ_ relaxation curves were sampled using 7–10 points for all experiments. 1–25 kHz nutation frequencies, measured by nutation experiments, were used for the spin-lock fields in the *R*_1ρ_ experiments (see SI table 13 for number of points used and total duration of the experiments). Residues which are far away from the center of the spectra in the ^15^N dimension and not covered by the spin-lock pulse were removed. These included; GB1_dia_ at 700 MHz ^1^H Larmor frequency and 1.1 kHz spin-lock frequency (6, 8, 9, 11, 12, 14, 19, 20, 38, 40, 41, 44, 46, 49, 52, 56), GB1_dia_ at 700 MHz ^1^H Larmor frequency and 1.5 kHz spin-lock frequency (11, 14, 40, 49, 52, 56), GB1_pre_ at 700 MHz ^1^H Larmor frequency and 1 kHz spin-lock frequency (2, 6, 8, 9, 11, 12, 14, 19, 40, 44, 46, 49, 52, 56) GB1_pre_ at 700 MHz ^1^H Larmor frequency and 1.5 kHz spin-lock frequency (11, 14, 40, 49, 52, 56), GB1_pre_ at 600 MHz ^1^H Larmor frequency and 1.2 kHz spin-lock frequency (11, 12, 14, 40, 49, 52, 56), GB1 in complex with IgG at 850 MHz ^1^H Larmor frequency and 1.85 kHz spin-lock frequency (11, 40, 49, 52, 56), GB1 in complex with IgG at 850 MHz ^1^H Larmor frequency and 2.45 kHz spin-lock frequency (11, 49), GB1 in complex with IgG at 700 MHz ^1^H Larmor frequency and 2 kHz spin-lock frequency (49, 56). All spectra were processed using TopSpin 3.2. GB1 resonances in the complex with IgG were previously assigned on the basis of 3D H(H)NH, CONH, CO(CA)NH and CANH experiments^4^. Peak integrals were calculated in TopSpin 3.2. OriginPro 2016 and MatLab R2014a were used to analyze the relaxation data.

### Data fitting

Peak integrals from TopSpin were exported to MatLab where a mono-exponential function was used with the fminsearchbnd function to fit the relaxation data. Average values calculated from integral regions containing only noise were used as input errors. Fit errors were calculated by Monte Carlo error estimations. A random number between 0 and 1 was multiplied with the integral error and added to the recalculated integrals. The fitting was then repeated 2000 times with a new random number between 0 and 1 generated each time. Two times the standard deviations of the *R*_1ρ_ values received from the fits for each residue were used as errors.

Exchange coefficients (*k*_ex_), populations of the minor state and major sites (*p*_A_*p*_B_), and difference in chemical shifts between the two states (Δ*δ*) were obtained by fitting the *R*_1ρ_ values obtained at two different *B*_0_ fields simultaneously to a Bloch-McConnell two-site exchange Eq. (1) derived as described in^37,38^. Since the populations and the chemical shift differences are highly correlated, the product of the two, *φ*_ex_, was extracted from the fits instead.

1 $${R}_{1\rho }={R}_{1\rho ,0}+\frac{{p}_{A}{p}_{B}{\rm{\Delta }}{\delta }^{2}{k}_{ex}}{{\omega }_{1}^{2}+{k}_{ex}^{2}}$$

where *R*_1ρ,0_ is the plateau value for *R*_1ρ_ and *ω*_1_ is the ^15^N spin-lock field strength. For the higher *B*_0_ field used in the fits the ratio between the fields squared was multiplied with the fraction in the equation to account for the differences in field strengths. Errors were calculated using Monte Carlo error estimation in the same way as for *R*_1ρ_ exponential fits but with 250 repeats of the fitting. In these fits, on resonance *R*_1ρ_ rates were used, and were calculated from the measured rates (*R*_1ρ,obs_) by Eq. 2:

2 $${R}_{1\rho }=\frac{{R}_{1\rho ,obs}-{\cos }^{2}\theta {R}_{1}}{{\sin }^{2}\theta }$$

where the *R*_1_ rates used are published elsewhere^3,39^ and the angle θ was calculated from Eq. 3 with Ω as the offset for each peak.

3 $$\theta ={\tan }^{-1}\frac{{\omega }_{1}}{\Omega }$$

For modelling of spinning frequency dependent ^15^N *R*_1ρ_, both, dipolar NH (*r*_NH_ = 1.02 Å) and ^15^N chemical shift anisotropy (CSA (assuming axially symmetric CSA tensor collinear with the NH vector; ${\rm{\Delta }}\sigma ={\sigma }_{\parallel }-{\sigma }_{\perp }$ = −170 ppm, η = 0) contributions, were considered.

The dipolar contribution was expressed as^3,14^

4 $$\begin{array}{rcl}{R}_{1\rho ,NH} & = & \frac{1}{20}{(\frac{{\mu }_{0}}{4\pi }\frac{\hslash {\gamma }_{H}{\gamma }_{N}}{{r}_{NH}^{3}})}^{2}(\frac{2}{3}J\,({\omega }_{1}+2{\omega }_{r})+\frac{2}{3}J\,({\omega }_{1}-2{\omega }_{r})+\frac{4}{3}J\,({\omega }_{1}+{\omega }_{r})+\frac{4}{3}J\,({\omega }_{1}-{\omega }_{r})+3J\,({\omega }_{N})\\  &  & +\,J\,({\omega }_{H}-{\omega }_{N})+6J\,({\omega }_{H})+6J\,({\omega }_{H}+{\omega }_{N}))\end{array}$$

and CSA contribution

5 $${R}_{1\rho ,NCSA}=\frac{1}{45}{({\sigma }_{\parallel }-{\sigma }_{\perp })}^{2}(\frac{2}{3}J\,({\omega }_{1}+2{\omega }_{r})+\frac{2}{3}J\,({\omega }_{1}-2{\omega }_{r})+\frac{4}{3}J\,({\omega }_{1}+{\omega }_{r})+\frac{4}{3}J\,({\omega }_{1}-{\omega }_{r})+3J\,({\omega }_{N}))$$

where *ω*_*r*_ is the spinning frequency, *ω*_1_ is the spin lock nutation frequency, and *ω*_*N*_, *ω*_*H*_ the nitrogen and proton Larmor frequencies, respectively.

The expression for the order parameter of the two-site jump model was^54^

6 $${S}^{2}=\sum _{i,j}\frac{{p}_{i}{p}_{j}}{2}(3{\cos }^{2}\theta -1)$$

where *p*_i_, *p*_j_ are the populations of the two states and *θ* is the jump angle.

The simple model free spectral density was defined as

7 $$J(\omega )=(1-{S}^{2})\frac{\tau }{1+{(\omega \tau )}^{2}}$$

The fitting of the data was done by minimization of the χ^2^ target function:

8 $${\chi }^{2}=\sum \frac{{({X}_{i,calc}-{X}_{i,\exp })}^{2}}{{\sigma }_{i,\exp }^{2}}$$

where χ_i_ are the data sets and σ_i_ the corresponding error.

In fits where data from two magnetic fields were combined the minimization of the χ^2^ target functions for both magnetic fields was performed simultaneously. To adjust for the different number of spin-lock fields used for crystalline GB1 at different magnetic fields, a weighting factor was used so that data from each magnetic field is contributing equally to the final χ^2^ value. The weighting factor was determined by comparing the minimum χ^2^ value obtained by fitting data from each magnetic field separately (see Supplementary Fig. S15).

To compare how different models performed, the Bayesian Information Criterion (BIC) was calculated for each model as:

9 $$BIC={\chi }^{2}+k\,\mathrm{ln}(n)$$

where *k* is the number of fit parameters and *n* the number of data points (see Supplementary Table S10 for comparisons between different models).

## Supplementary information

Supplementary Information

## Data Availability

Raw NMR data is available on WRAP at the link: http://wrap.warwick.ac.uk/117521/.
