# Supplementary material for: Quantifying Microsecond Exchange in Large Protein Complexes with Accelerated Relaxation Dispersion Experiments in the Solid State
Source: Sci Rep. 2019 Jul 31;9:11082. doi: 10.1038/s41598-019-47507-8 (PMC6668460; doi:10.1038/s41598-019-47507-8)
Supplement: Supplementary file 1 — Supplementary Information [file 41598_2019_47507_MOESM1_ESM.docx]

Supplementary Information for

**Quantifying Microsecond Exchange in Large Protein Complexes with Accelerated Relaxation Dispersion Experiments in the Solid State**

Carl Öster, Simone Kosol and Józef R Lewandowski*

Department of Chemistry, University of Warwick, Gibbet Hill Road, Coventry, CV4 7AL, U.K.


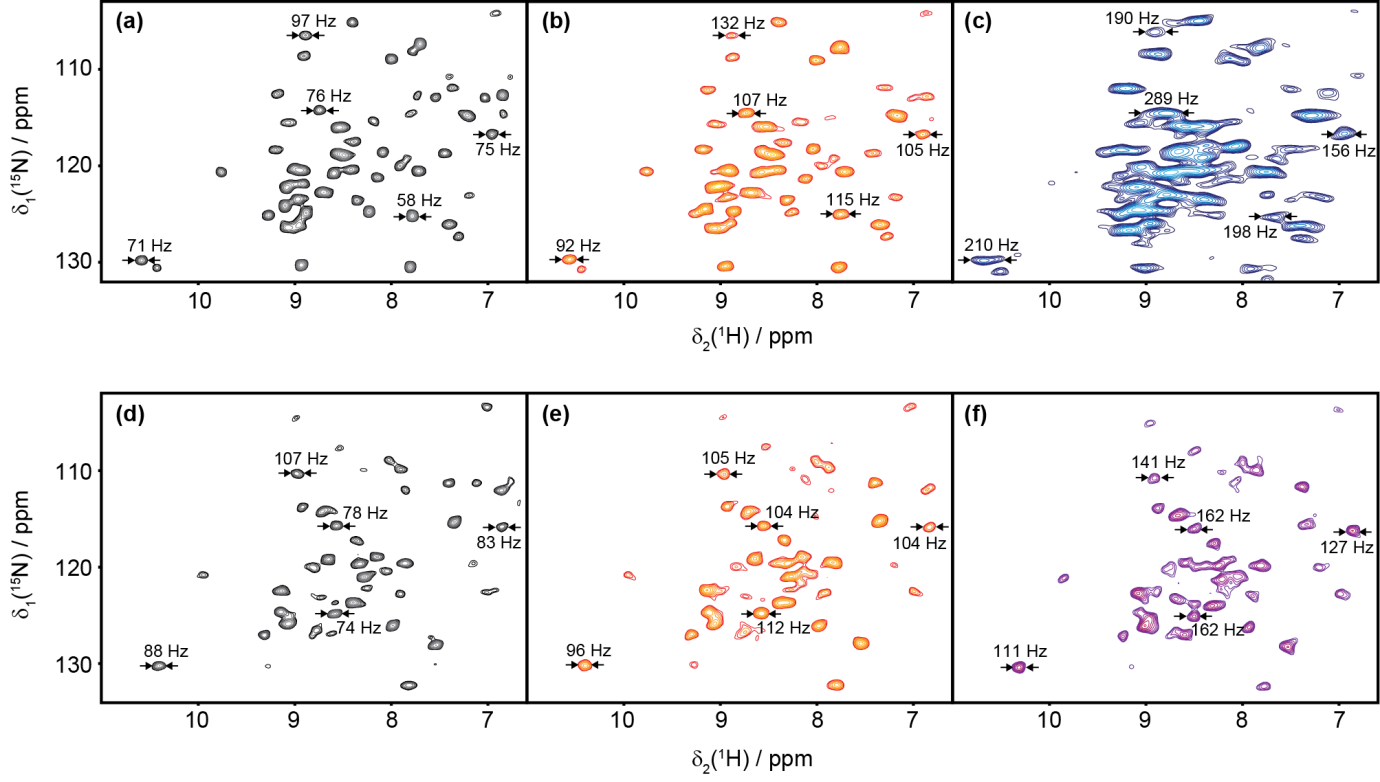


**Figure S1.** Examples of ^1^H-^15^N 2D correlation spectra and representative ^1^H linewidths of samples containing different concentrations of Gd(DTPA-BMA) as paramagnetic relaxation enhancement agent. All experiments were acquired at 60 kHz MAS with a sample temperature of 300 ± 2 K. (a) Spectrum of crystalline GB1 without any paramagnetic agent at 700 MHz ^1^H Larmor frequency. (b) Spectrum of crystalline GB1 with 2mM Gd(DTPA-BMA) at 700 MHz ^1^H Larmor frequency. (c) Spectrum of crystalline GB1 with 4mM Gd(DTPA-BMA) at 600 MHz ^1^H Larmor frequency. (d) Spectrum of GB1 in complex with IgG without any paramagnetic agent at 700 MHz ^1^H Larmor frequency. (e) Spectrum of GB1 in complex with IgG with 2 mM Gd(DTPA-BMA) at 700 MHz ^1^H Larmor frequency. (f) Spectrum of GB1 in complex with IgG with 5 mM Gd(DTPA-BMA) at 850 MHz ^1^H Larmor frequency.


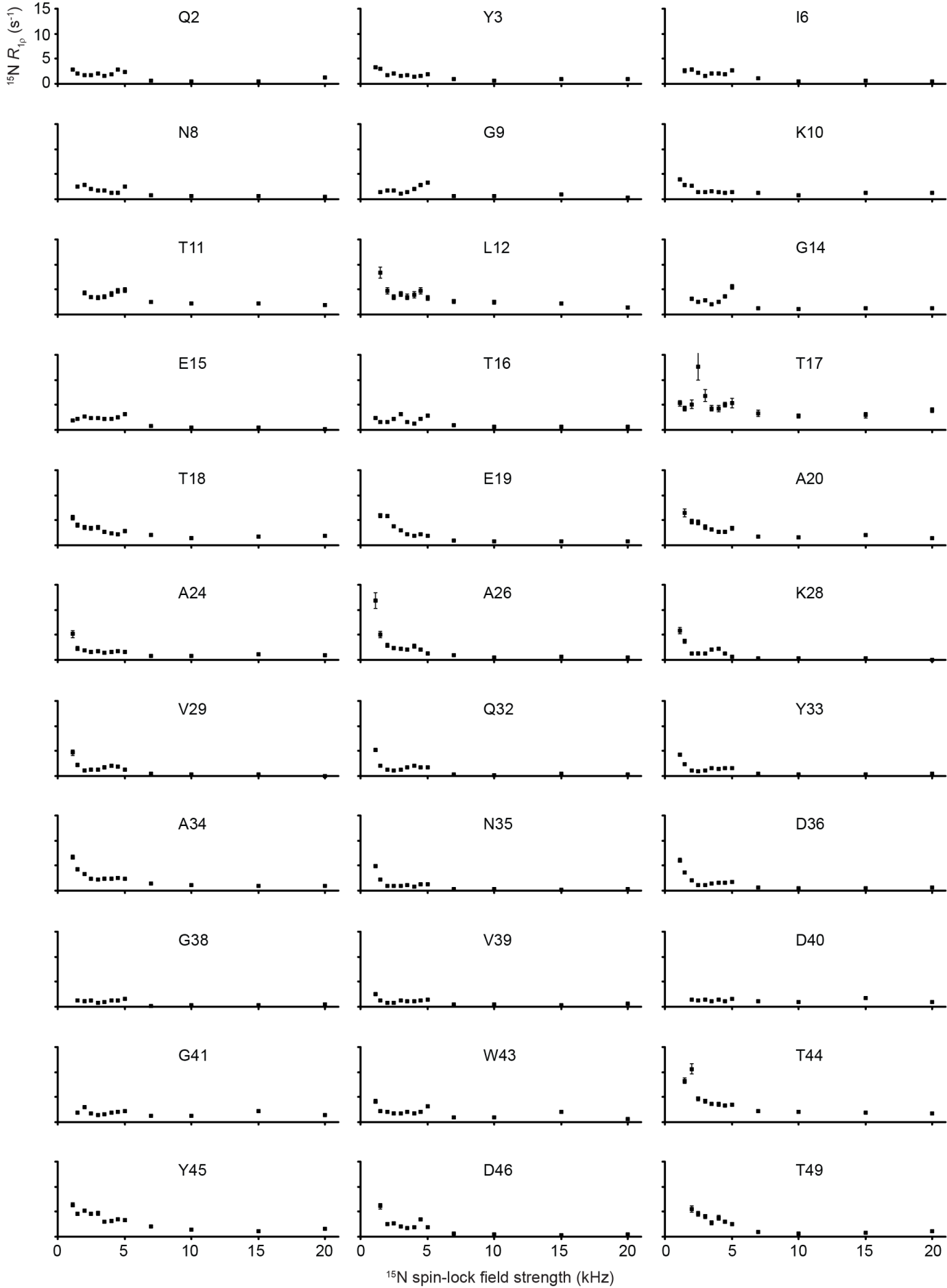


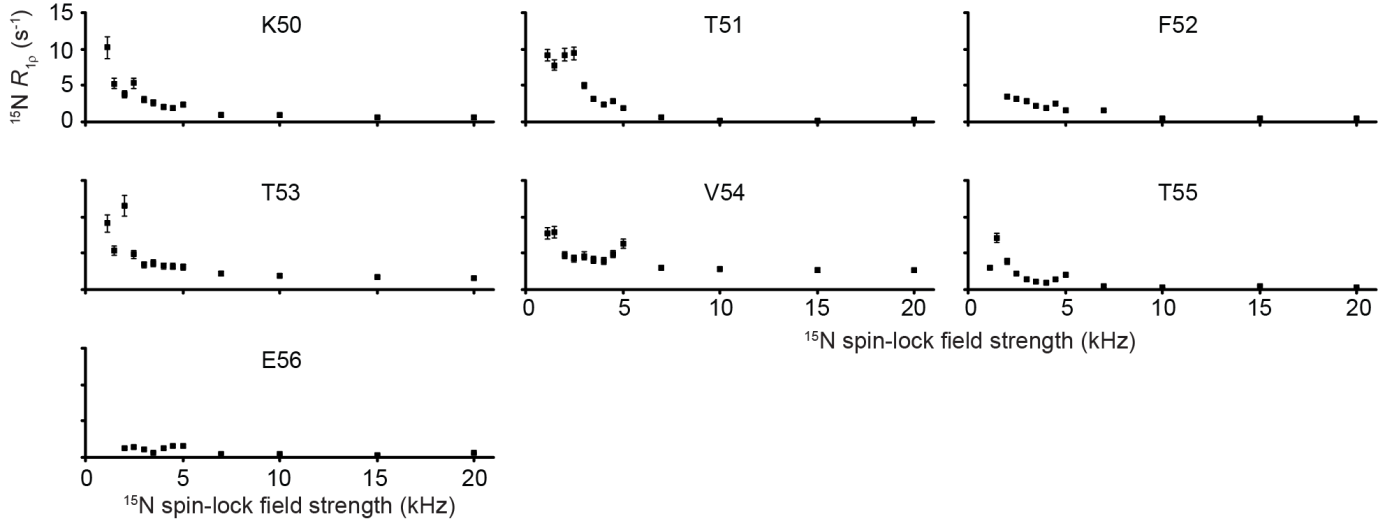


**Figure S2.** Relaxation dispersion profiles for GB1_dia_ at 700 MHz ^1^H Larmor frequency (16.4 T). The values on the y-axes are the same as the first plot for all plots and the values on the x-axes are the same as in the last plot for all.


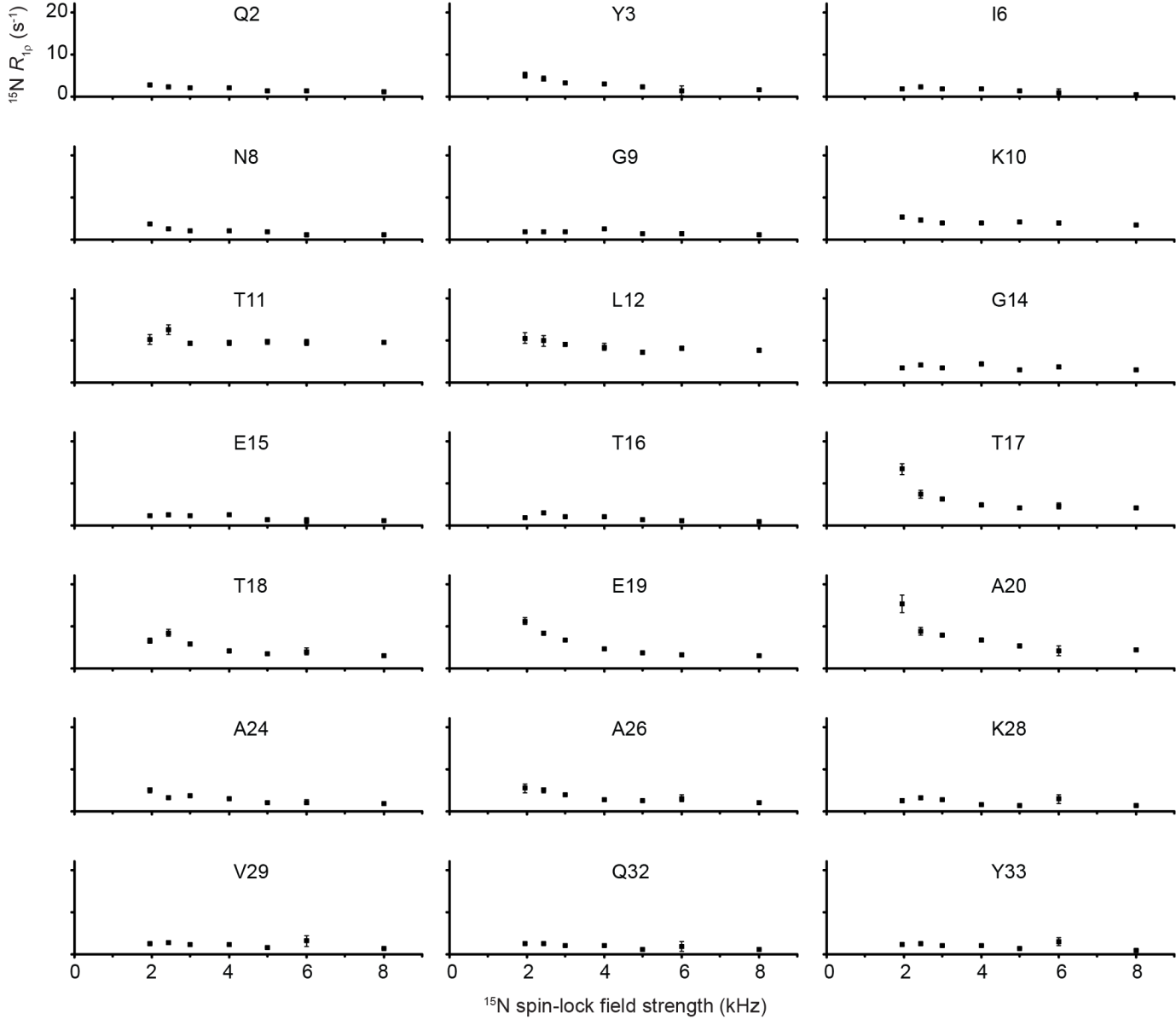


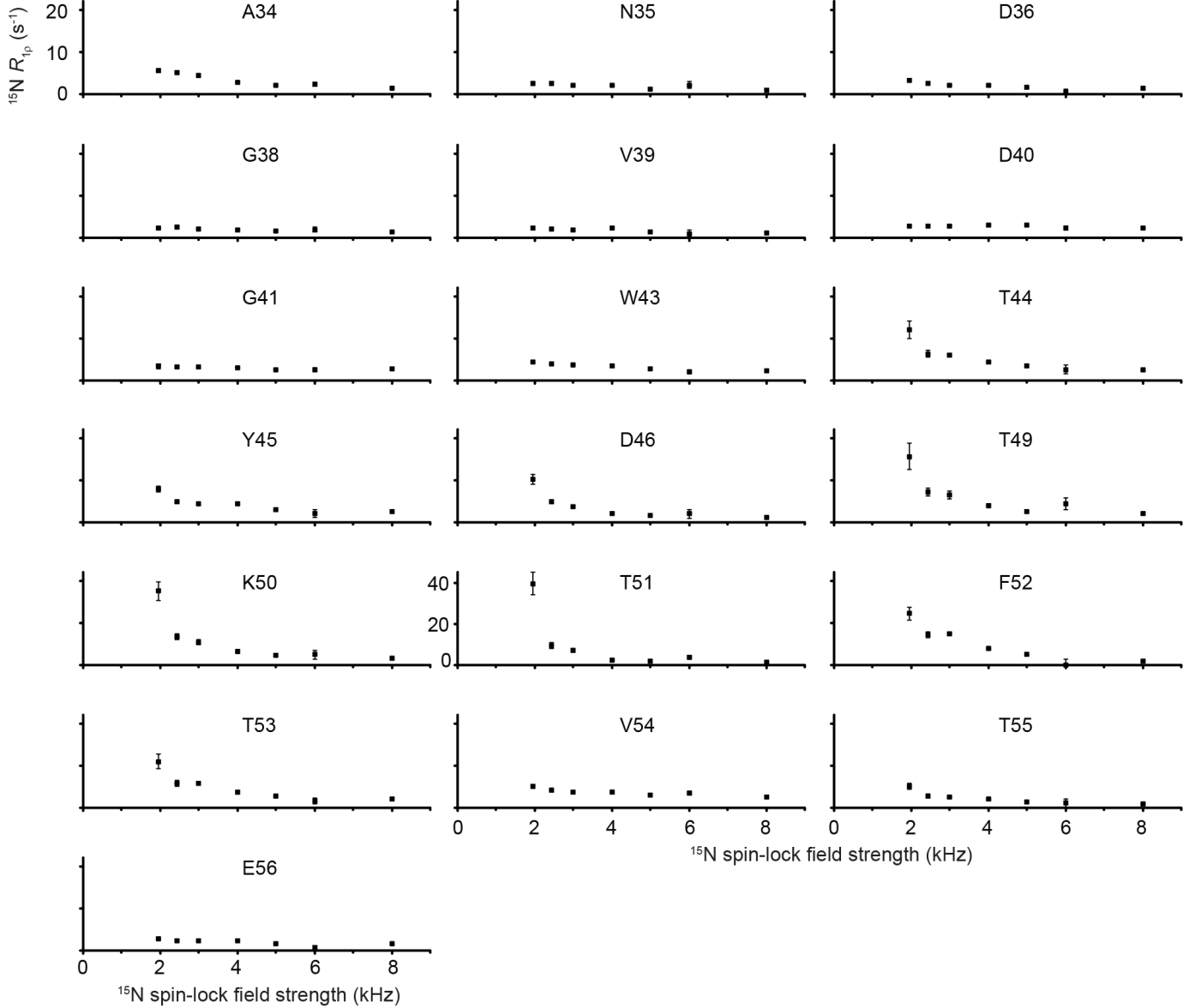


**Figure S3**. Relaxation dispersion profiles for GB1_dia_ at 600 MHz ^1^H Larmor frequency (14.1 T). Based on previously published data^1^. The values on the y-axes are the same as the first plot for all plots and the values on the x-axes are the same as in the last plot for all.


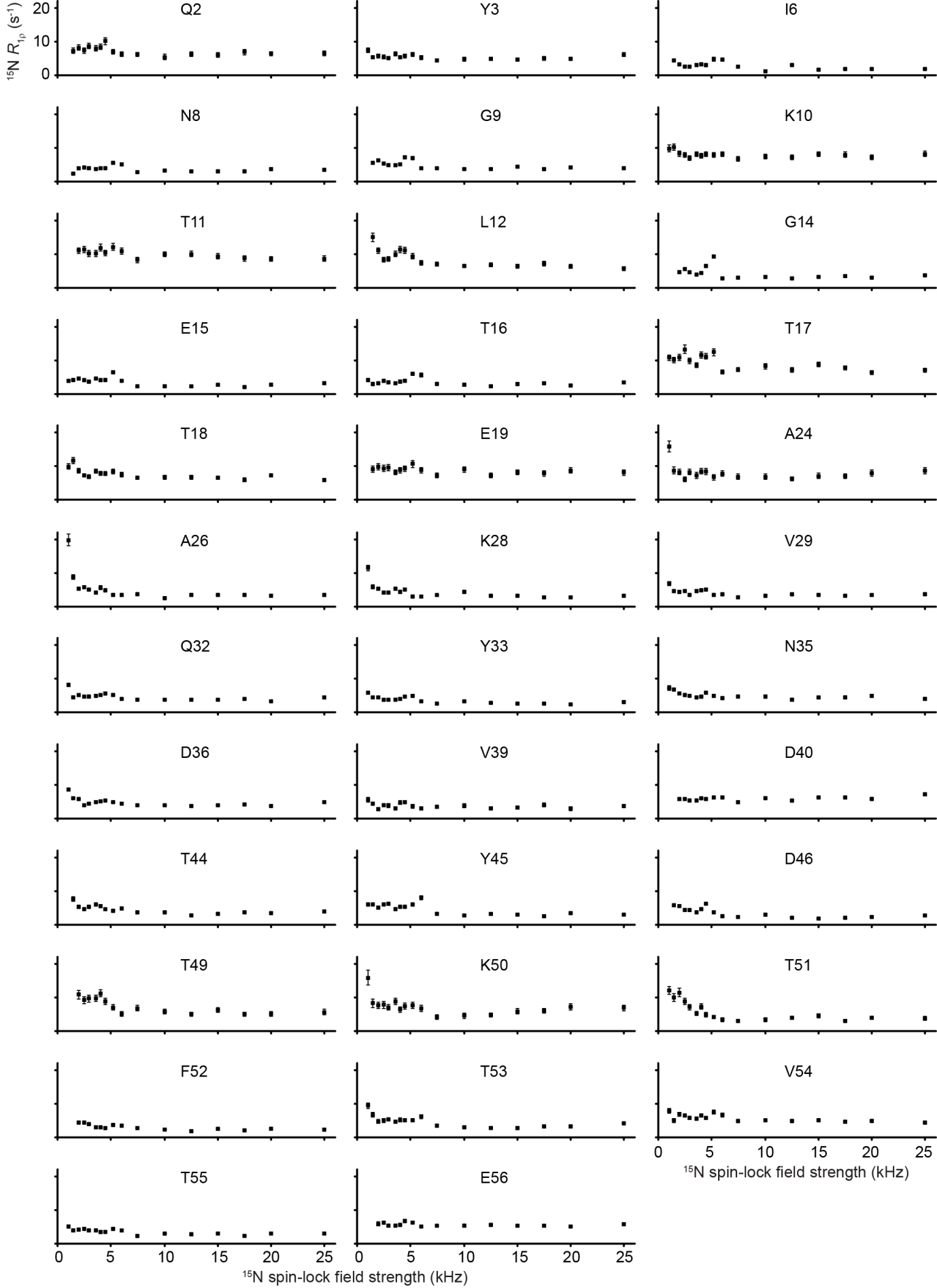


**Figure S4**. Relaxation dispersion profiles for GB1_pre_ at 700 MHz ^1^H Larmor frequency (16.4 T). The values on the y-axes are the same as the first plot for all plots and the values on the x-axes are the same as in the last plot for all.


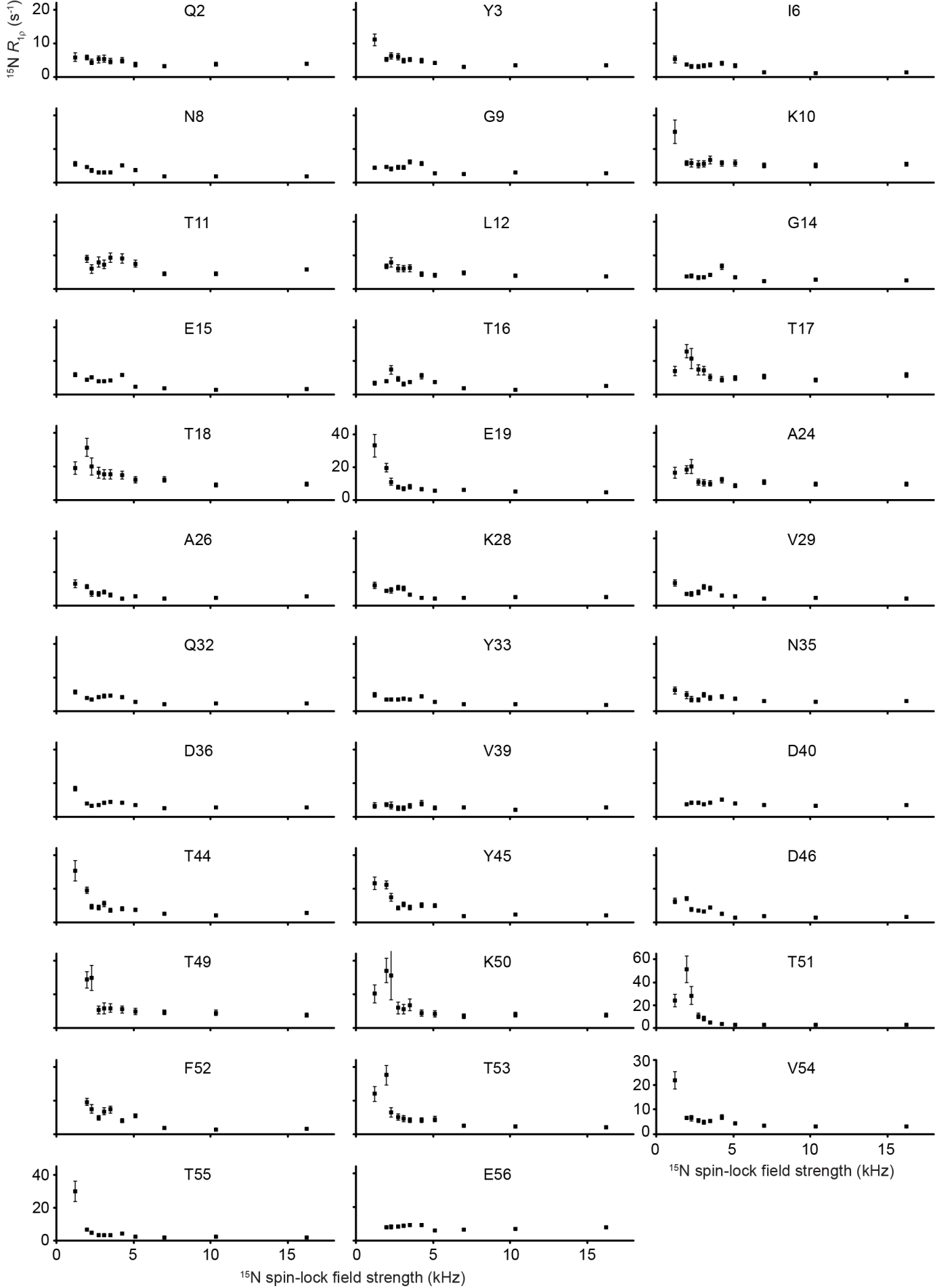


**Figure S5**. Relaxation dispersion profiles for GB1_pre_ at 600 MHz ^1^H Larmor frequency (14.1 T). The values on the y-axes are the same as the first plot for all plots except for E19, T51, V54 and T55. The values on the x-axes are the same as in the last plot for all.


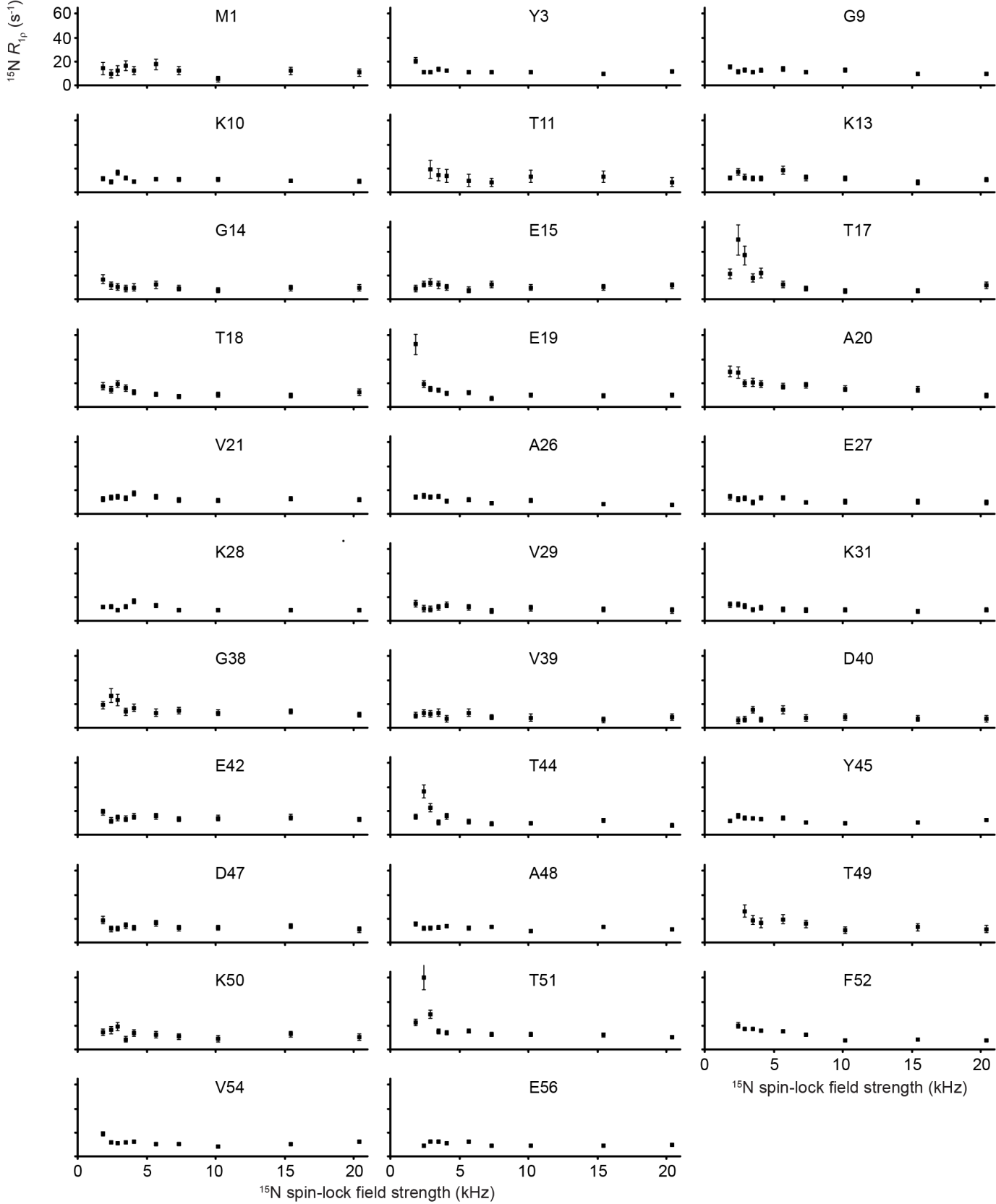


**Figure S6**. Relaxation dispersion profiles for GB1 in complex with IgG, with 5 mM Gd(DTPA-BMA) as paramagnetic relaxation enhancement agent, measured at 850 MHz ^1^H Larmor frequency (20 T). The values on the y-axes are the same as the first plot for all plots and the values on the x-axes are the same as in the last plot for all.


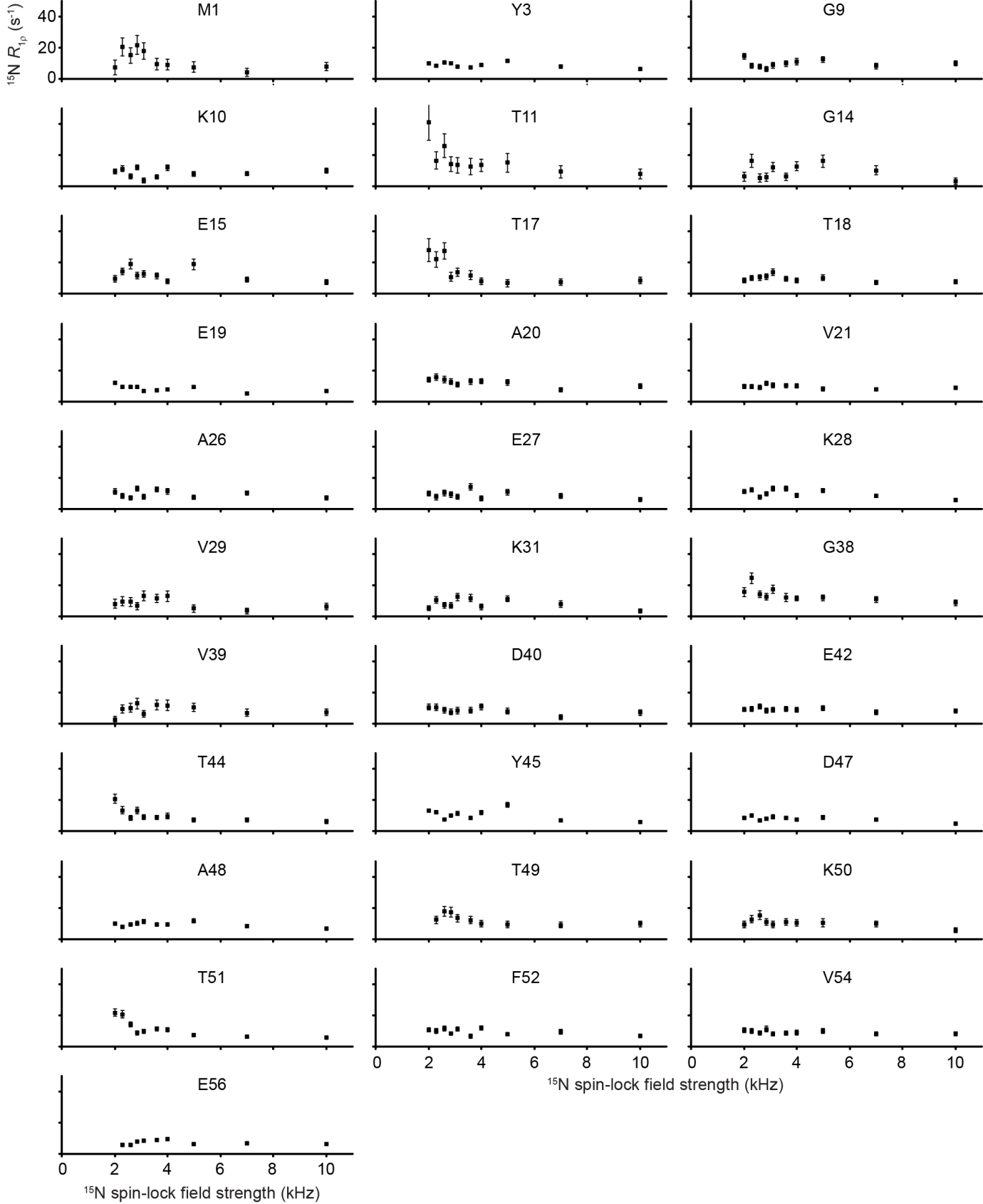


**Figure S7.** Relaxation dispersion profiles for GB1 in complex with IgG, with 2 mM Gd(DTPA-BMA) as paramagnetic relaxation enhancement agent, measured at 700 MHz ^1^H Larmor frequency (16.4 T). The values on the y-axes are the same as the first plot for all plots and the values on the x-axes are the same as in the last plot for all.


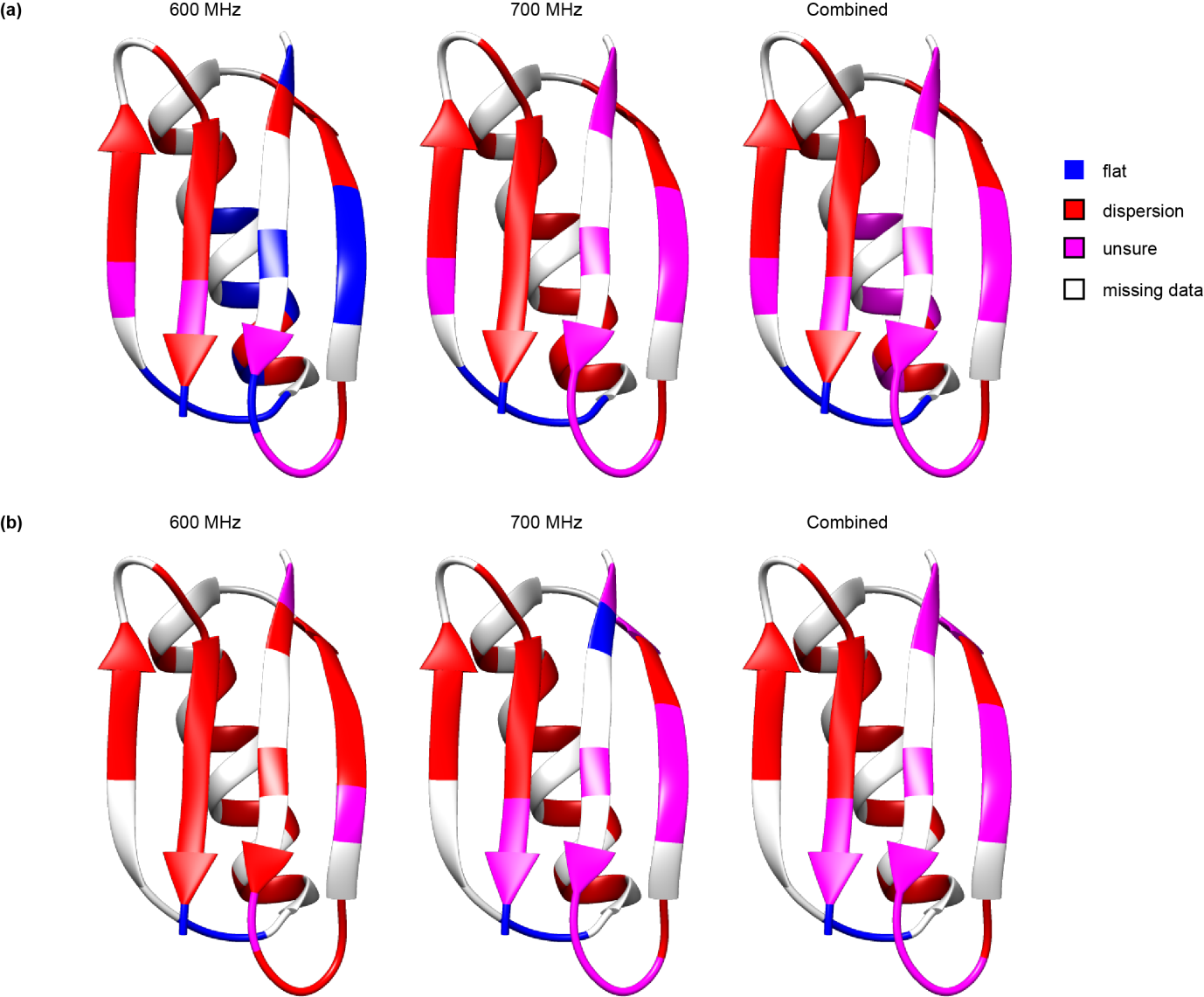


**Figure S8**. GB1 structures (PDB ID: 2QMT^2^) with residues colored based on relaxation dispersion profiles. (a) GB1_dia_. (b) GB1_pre_. Residues showing flat dispersion curves are blue, residues showing dispersion are red, residues that couldn’t be clearly identified as being flat or showing dispersion are pink and residues for which data are missing due to severe overlap or missing peaks in the spectra are grey.


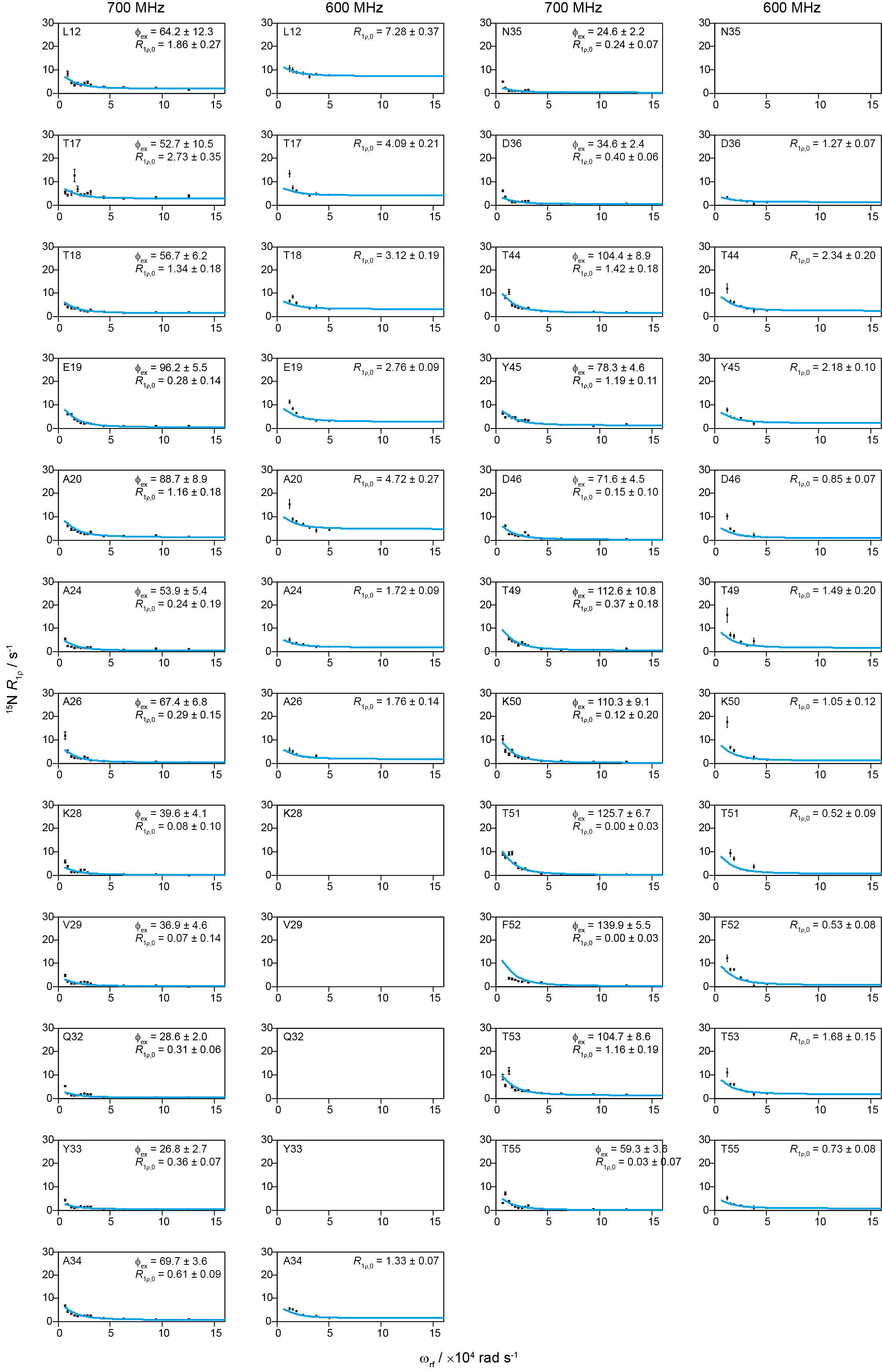


**Figure S9.** Relaxation dispersion fits based on data from GB1_dia_ (blue lines, *k*_ex_ = 14340 ± 540 s^-1^) obtained from measurements at 600 MHz (14.1 T) and 700 MHz (16.4 T) and a sample temperature of 300 ± 2 K. Residue specific *ϕ*_ex_ and *R*_1ρ,0_ are indicated in each plot.


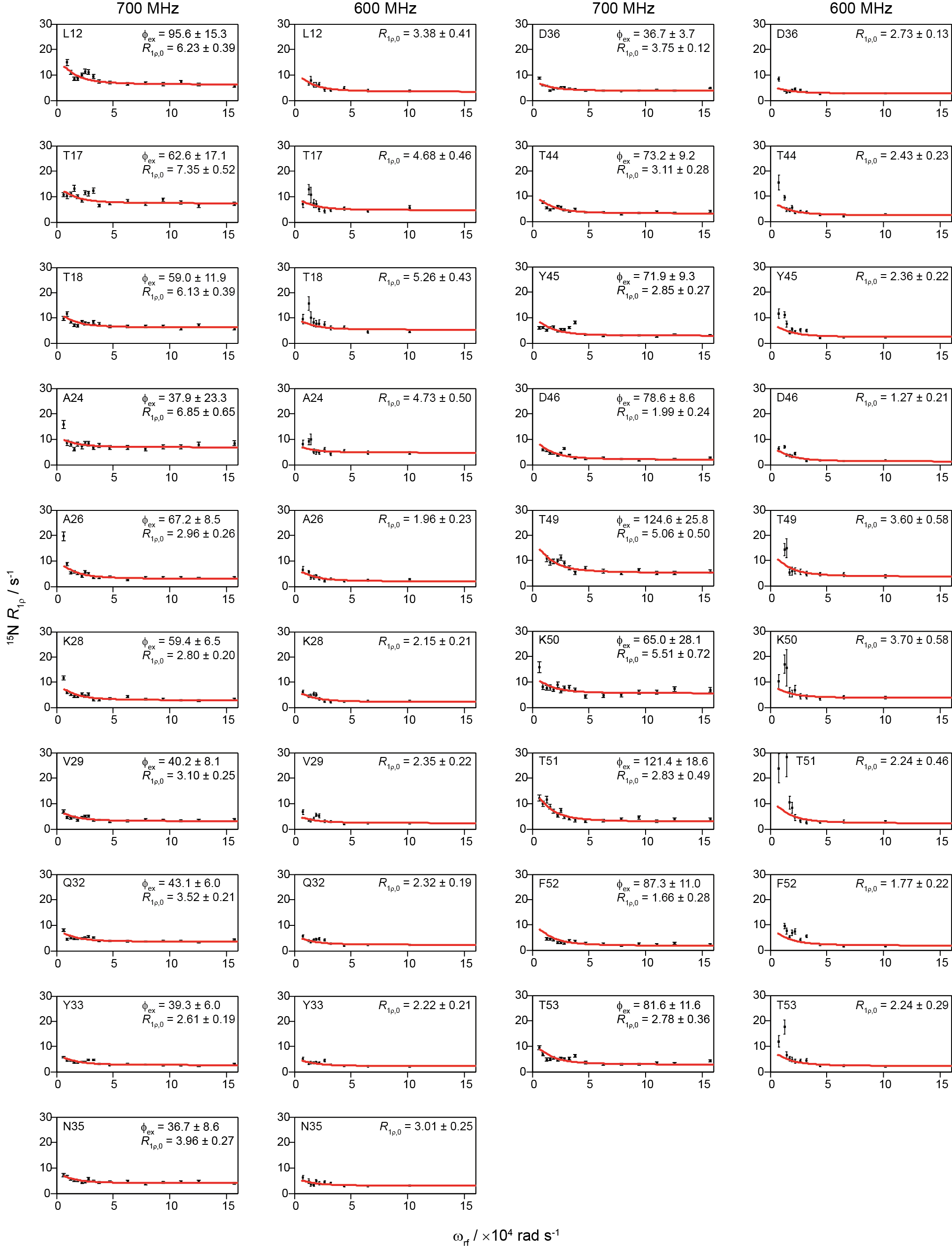


**Figure S10.** Relaxation dispersion fits based on data from GB1_pre_ (red lines, *k*_ex_ = 15480 ± 2120 s^-1^) obtained from measurements at 600 MHz (14.1 T) and 700 MHz (16.4 T) and a sample temperature of 300 ± 2 K. Residue specific *ϕ*_ex_ (×10^3^ rad^2^ s^-2^) and *R*_1ρ,0_ (s^-1^) are indicated in each plot.


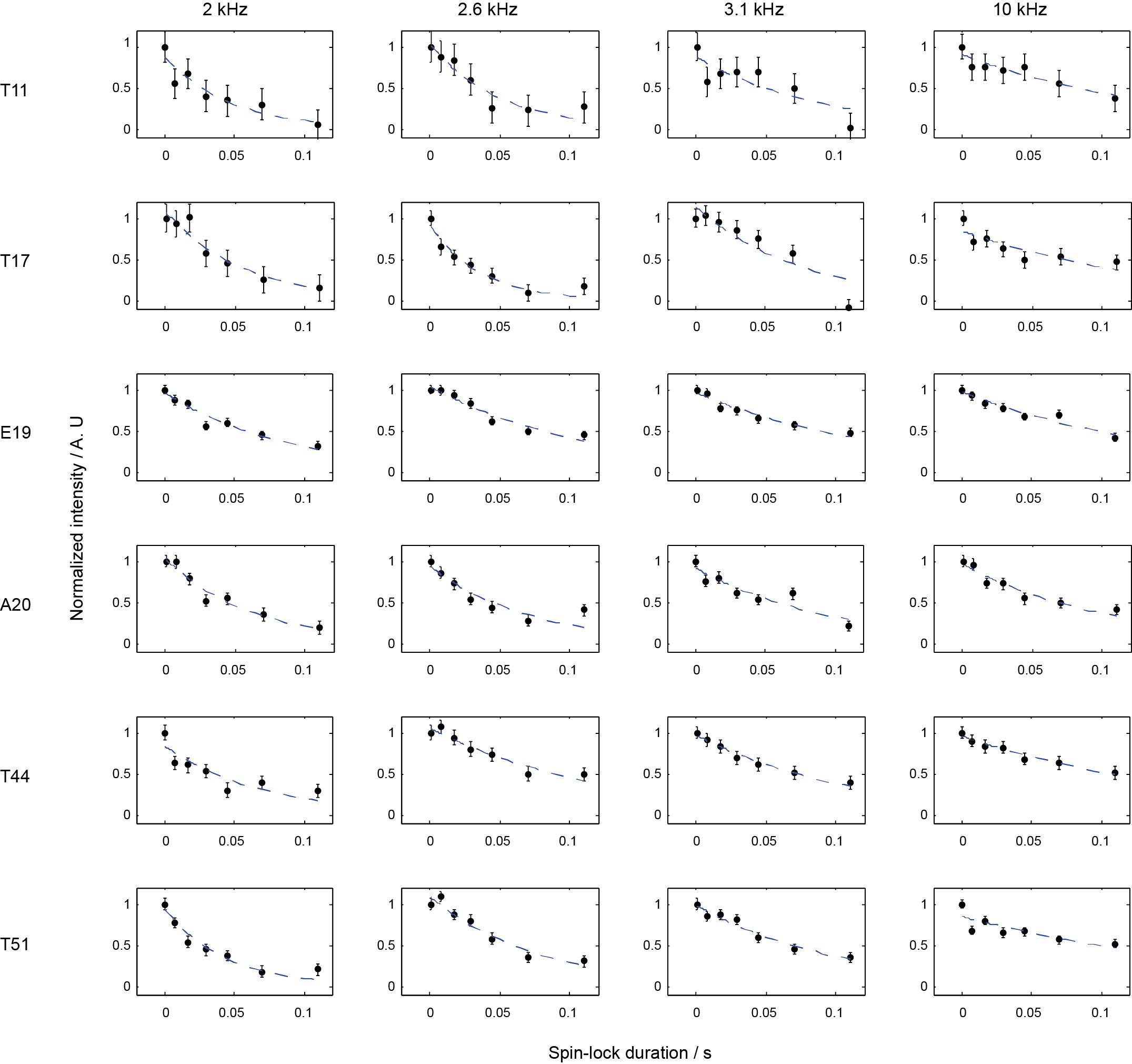


**Figure S11.** Examples of *R*_1ρ_ fits for residues with varying signal to noise ratios of GB1 in complex with IgG obtained from measurements with different spin-lock fields at 700 MHz (16.4 T) and a sample temperature of 300 ± 2 K.


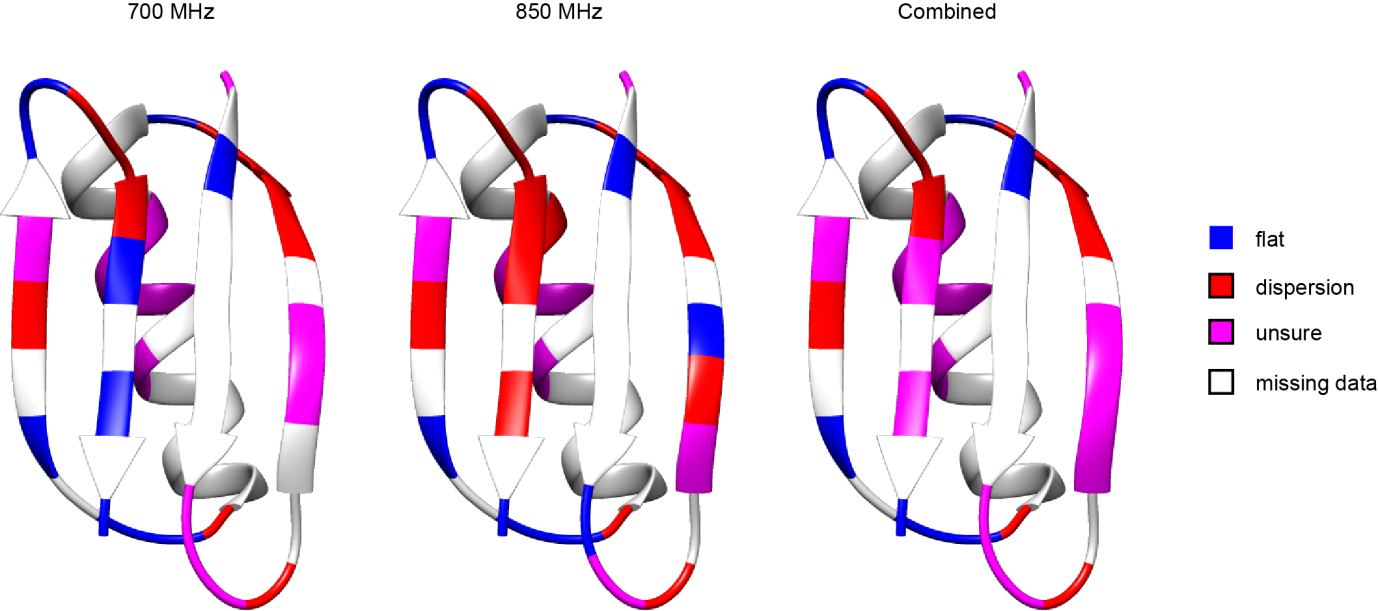


**Figure S12**. GB1 structures (PDB ID: 2QMT^2^) with residues colored based on relaxation dispersion profiles of GB1 in complex with IgG. Residues showing flat curves are blue, residues showing dispersion are red, residues that couldn’t be clearly identified as being flat or showing dispersion are pink and residues for which data are missing due to severe overlap or missing peaks in the spectra are grey.


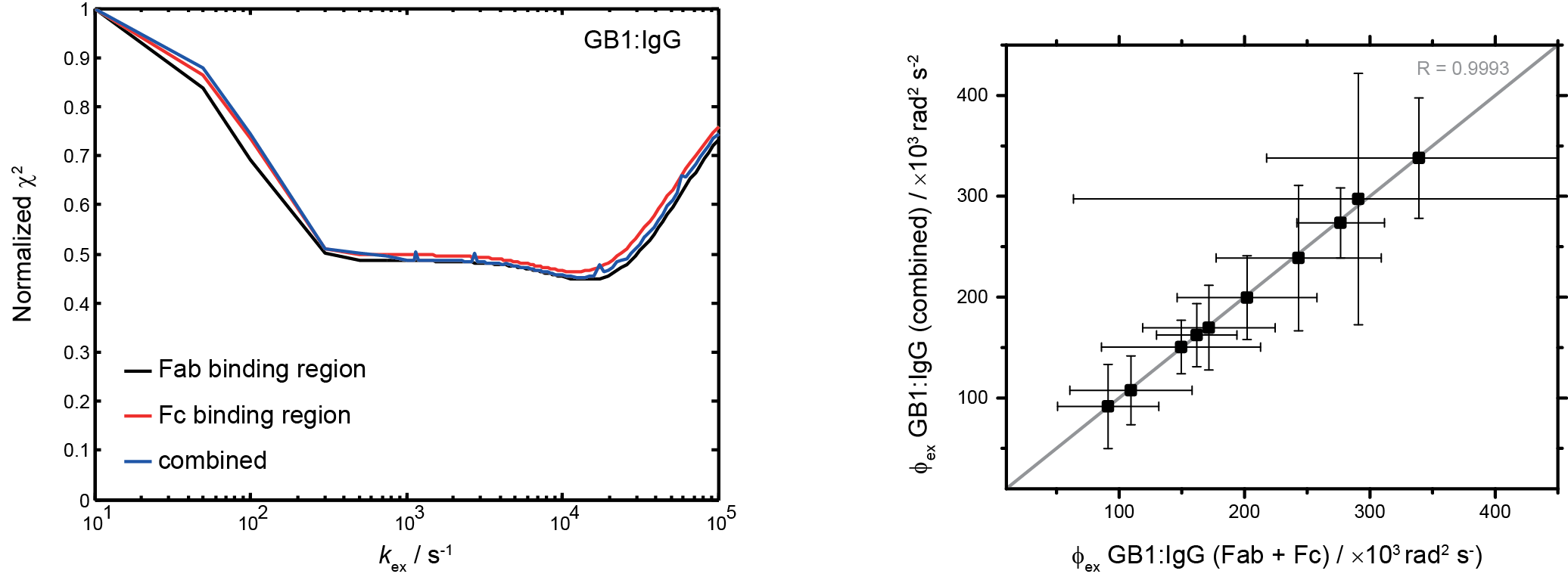


**Figure S13**. Comparison between fits of all residues together and separate fits for the Fc and Fab binding regions of GB1 in complex with IgG. Agreement between experimental and fitted data represented by plots between fixed exchange coefficients, *k*_ex_, and the corresponding χ^2^ values for different groupings of GB1 in complex with IgG (left). Correlation plots for *ϕ*_ex_ values based on fits of all residues together and the Fc and Fab binding regions separately (right).


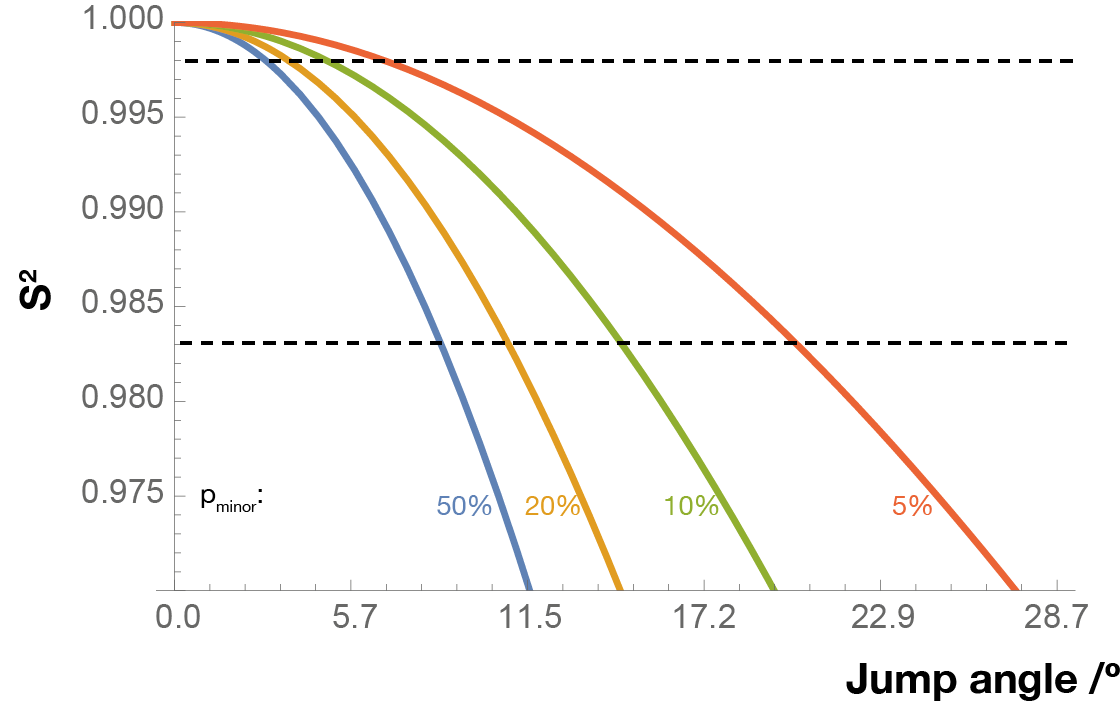


**Figure S14**. Order parameter for a two-site jump model with unequal populations as a function of the jump angle. Curves for minor populations of 5, 10, 20 and 50% are shown. The dashed lines indicate the range of order parameters determined from simultaneous fitting of the data obtained for crystalline GB1 at MAS rates of 39, 50 and 56 kHz at a 600 MHz spectrometer and at 50 kHz and 60 kHz at a 700 MHz spectrometer, sample temperature ~300 K, to a simple model free formalism with the time scale fixed to 67 μs, which is the inverse of the average *k*_ex_ for GB1_dia_ and GB1_dia_ in the β-sheet region.


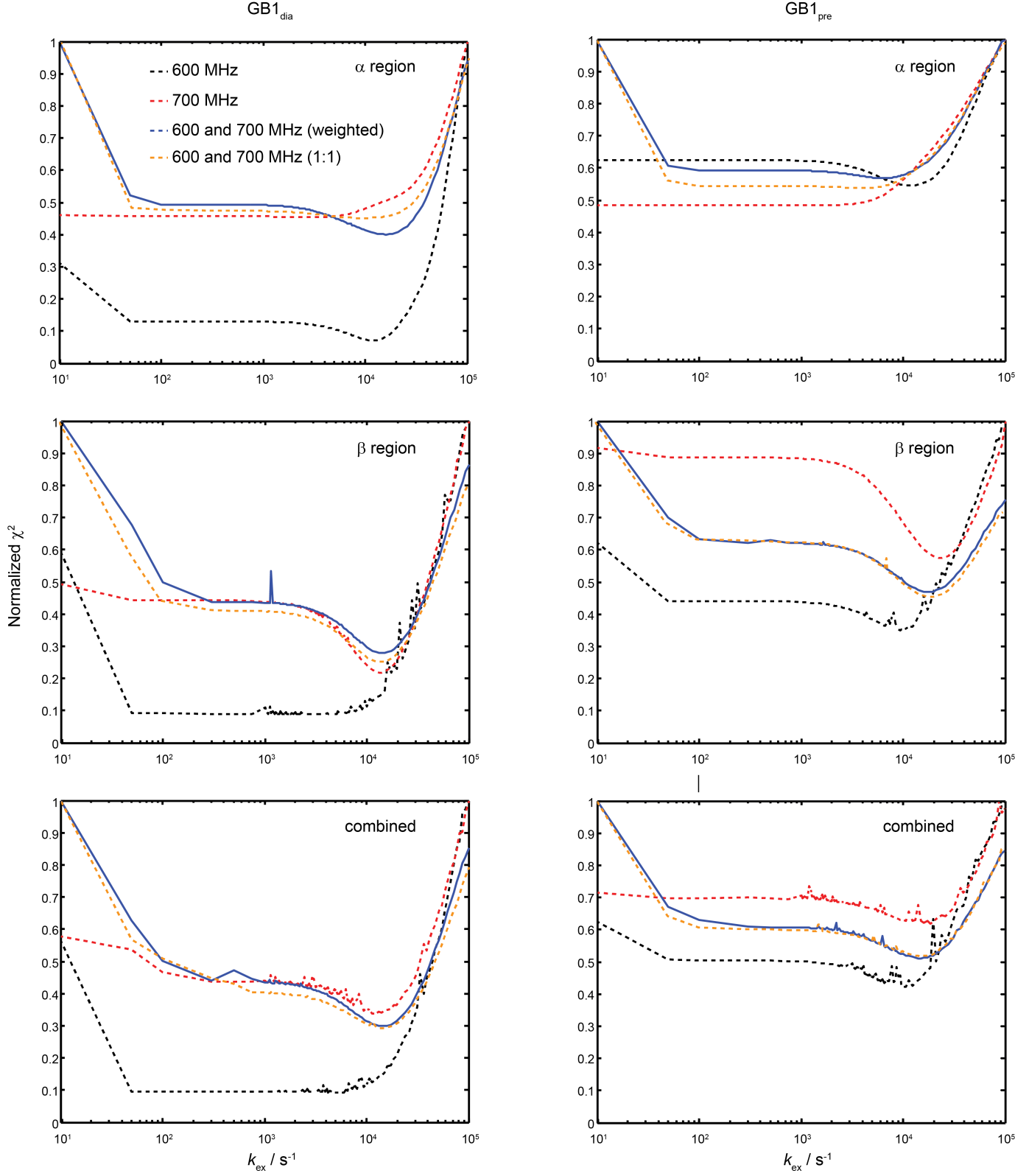


**Figure S15.** Comparison between fits of data acquired at a single external magnetic field (600 MHz black dotted lines, 700 MHz red dotted lines) and fits of data from both magnetic fields together with or without weighting factor (blue lines and dotted orange lines respectively). Agreement between experimental and fitted data is represented by plots between fixed exchange coefficients, *k*_ex_, and the corresponding χ^2^ values for different groupings of the residues in crystalline GB1. Fits for GB1_dia_ is shown in the left column and GB1_pre_ in the right column. The top row shows fits for the α region, the middle row shows fits for the β region and the bottom row shows combined fits for all residues showing dispersion. In the fits where data from two magnetic fields were combined the minimization of the χ^2^ target functions for both magnetic fields was performed simultaneously. To adjust for the different number of spin-lock fields used for crystalline GB1 at different magnetic fields a weighting factor was used so that data from each magnetic field is contributing equally to the final χ^2^ value. The weighting factors were determined to 3:1 (600 MHz:700 MHz) for GB1_dia_ and 2.5:1 (600 MHz:700 MHz) for GB1_pre_ by comparing the minimum χ^2^ value obtained from the fits based on each magnetic field separately.


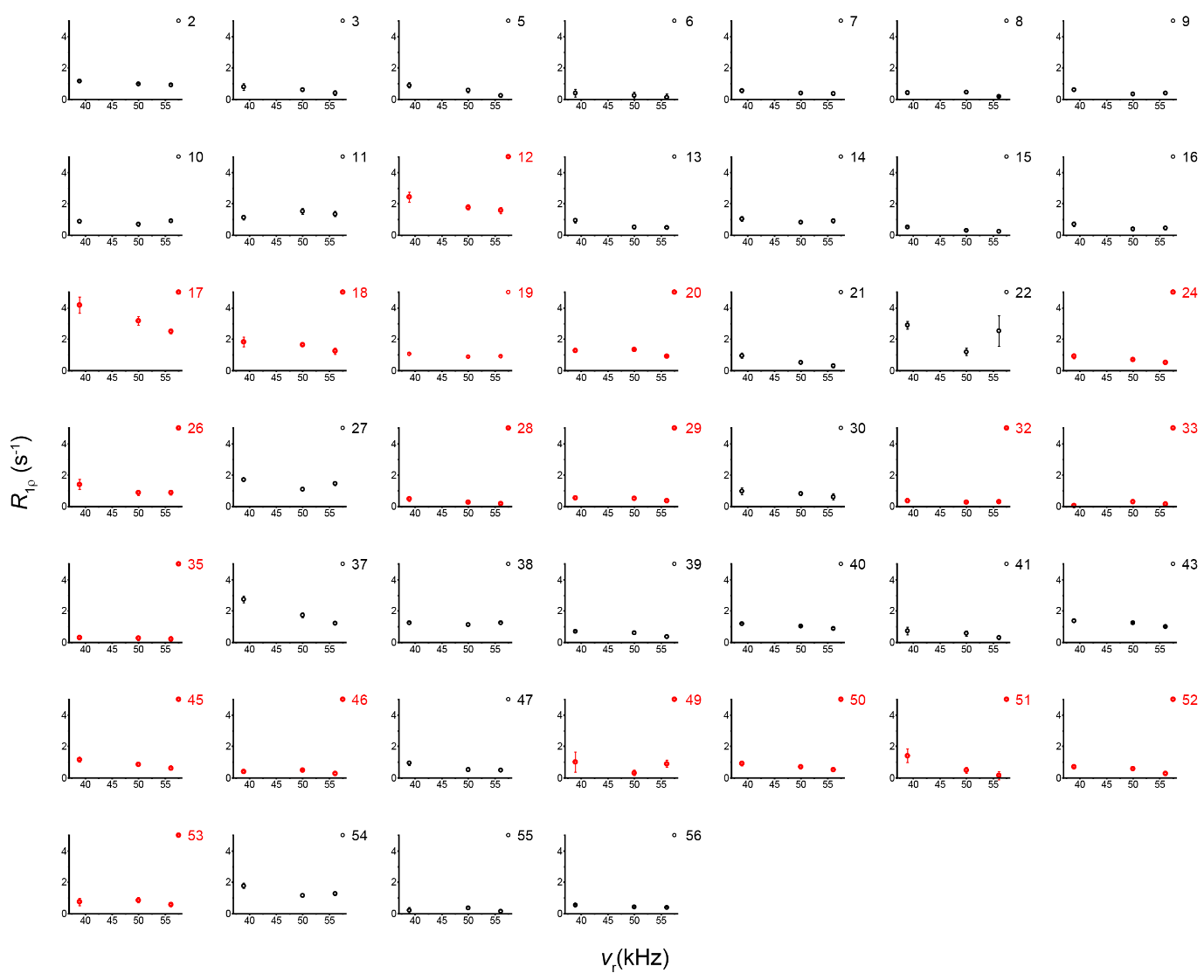


**Figure S16.** ^15^N *R*_1ρ_ as a function of spinning frequency for crystalline GB1 measured at a 600 MHz spectrometer and sample temperature of ~300 K. Data for residues, which exhibited clear relaxation dispersion in all data sets are shown in red.

**Table S1**. Comparison of signal to noise ratios (SNRs) per time unit for GB1:IgG samples with varying amounts of Gd (DTPA-BMA) based on 2D ^15^N - ^1^H spectra.

| Sample | 0 mM Gd | 1 mM Gd | 2 mM Gd | 3.5 mM Gd | 1 vs 0 mM | 2 vs 0 mM | 3.5 vs 0 mM |
| --- | --- | --- | --- | --- | --- | --- | --- |
| Residue | SNR per h | SNR per h | SNR per h | SNR per h | Increase (×) | Increase (×) | Increase (×) |
| 11 | 0.39 | 1.45 | 1.75 | 2.13 | 3.70 | 4.47 | 5.45 |
| 17 | 0.31 | 1.44 | 2.01 | 1.97 | 4.60 | 6.43 | 6.30 |
| 18 | 0.62 | 2.97 | 3.40 | 3.13 | 4.83 | 5.53 | 5.09 |
| 19 | 1.17 | 5.95 | 5.09 | 4.99 | 5.10 | 4.36 | 4.27 |
| 20 | 0.75 | 3.44 | 3.47 | 3.02 | 4.62 | 4.65 | 4.04 |
| 38 | 0.48 | 2.70 | 2.79 | 2.75 | 5.67 | 5.87 | 5.77 |
| 44 | 0.69 | 2.30 | 2.60 | 2.74 | 3.34 | 3.78 | 3.97 |
| 49 | 0.51 | 2.43 | 2.57 | 1.88 | 4.75 | 5.02 | 3.67 |
| 50 | 0.43 | 3.36 | 3.47 | 2.43 | 7.75 | 8.01 | 5.62 |
| 51 | 0.69 | 3.64 | 3.54 | 2.72 | 5.30 | 5.15 | 3.97 |
| Average increase in SNR / h (×) | | | |  | 4.97 | 5.33 | 4.82 |

**Table S2.** Results from RD fits of individual residues for GB1_dia_ at 14.1 and 16.4 T. The χ^2^ minimization failed for residues 33 and 35.

| Residue | *k*_ex_ (10^3^ s-^1^) | Error | ϕ_ex_ (10^3^ rad^2^ s-^2^) | Error | *R*_1ρ,0_ (s^-1^) at 16.4 T | Error | *R*_1ρ,0_ (s^-1^) at 14.1 T | Error |
| --- | --- | --- | --- | --- | --- | --- | --- | --- |
| 12 | 28.48 | 9.43 | 79.87 | 20.51 | 1.57 | 0.31 | 6.89 | 0.38 |
| 17 | 26.00 | 10.10 | 58.88 | 23.97 | 2.76 | 0.40 | 3.87 | 0.38 |
| 18 | 13.89 | 2.80 | 47.17 | 5.40 | 1.51 | 0.16 | 3.26 | 0.18 |
| 19 | 10.10 | 1.11 | 88.76 | 4.30 | 0.54 | 0.06 | 3.03 | 0.07 |
| 20 | 12.58 | 3.02 | 75.80 | 7.10 | 1.35 | 0.15 | 5.01 | 0.26 |
| 24 | 19.66 | 4.28 | 45.30 | 6.23 | 0.40 | 0.14 | 1.73 | 0.12 |
| 26 | 7.90 | 2.09 | 69.70 | 9.75 | 0.49 | 0.10 | 2.01 | 0.12 |
| 28 | 4.72 | 1.86 | 53.90 | 17.70 | 0.21 | 0.06 | 7.74 | 1.46 |
| 29 | 31.09 | 5.57 | 48.77 | 5.69 | 0.00 | 0.05 | 0.61 | 3.89 |
| 32 | 22.38 | 3.21 | 34.53 | 3.58 | 0.19 | 0.06 | 2.83 | 4.77 |
| 33 | 1.00 | 1.61 | 125.38 | 67.04 | 0.53 | 0.05 | 11.12 | 9.13 |
| 34 | 16.86 | 1.62 | 59.13 | 2.86 | 0.75 | 0.07 | 1.38 | 0.07 |
| 35 | 1.00 | 1.53 | 131.60 | 70.09 | 0.36 | 0.04 | 5.72 | 12.52 |
| 36 | 2.00 | 1.49 | 99.35 | 92.99 | 0.57 | 0.03 | 1.50 | 0.05 |
| 44 | 10.68 | 1.98 | 98.55 | 7.60 | 1.64 | 0.13 | 2.61 | 0.17 |
| 45 | 21.38 | 2.13 | 85.22 | 5.86 | 1.02 | 0.10 | 1.95 | 0.12 |
| 46 | 14.67 | 1.98 | 62.61 | 2.93 | 0.26 | 0.07 | 0.93 | 0.07 |
| 49 | 13.00 | 2.96 | 106.79 | 9.32 | 0.47 | 0.12 | 1.60 | 0.20 |
| 50 | 14.37 | 2.22 | 93.09 | 6.06 | 0.27 | 0.13 | 1.19 | 0.13 |
| 51 | 12.32 | 0.90 | 126.53 | 5.08 | 0.00 | 0.05 | 0.63 | 0.08 |
| 52 | 20.04 | 2.14 | 103.40 | 3.92 | 0.00 | 0.06 | 0.66 | 0.09 |
| 53 | 15.63 | 2.55 | 87.79 | 7.01 | 1.34 | 0.16 | 1.79 | 0.16 |
| 55 | 15.94 | 1.67 | 53.19 | 3.08 | 0.08 | 0.07 | 0.77 | 0.09 |

**Table S3**. Results from RD fits of all residues combined for GB1_dia_ at 14.1 and 16.4 T.

| Residue | *k*_ex_ (10^3^ s-^1^) | Error |  |  |  |  |
| --- | --- | --- | --- | --- | --- | --- |
| All | 14.34 | 0.54 |  |  |  |  |
|  | ϕ_ex_ (10^3^ rad^2^ s-^2^) | Error | *R*_1ρ,0_ (s^-1^) at 16.4 T | Error | *R*_1ρ,0_ (s^-1^) at 14.1 T | Error |
| 12 | 64.15 | 12.25 | 1.86 | 0.27 | 7.28 | 0.37 |
| 17 | 52.68 | 10.53 | 2.73 | 0.35 | 4.09 | 0.21 |
| 18 | 56.67 | 6.16 | 1.34 | 0.18 | 3.12 | 0.19 |
| 19 | 96.18 | 5.51 | 0.28 | 0.14 | 2.76 | 0.09 |
| 20 | 88.69 | 8.90 | 1.16 | 0.18 | 4.72 | 0.27 |
| 24 | 53.88 | 5.45 | 0.24 | 0.19 | 1.72 | 0.09 |
| 26 | 67.38 | 6.78 | 0.29 | 0.15 | 1.76 | 0.14 |
| 28 | 39.57 | 4.09 | 0.08 | 0.10 | 0.01 | 0.54 |
| 29 | 36.95 | 4.61 | 0.07 | 0.14 | 0.03 | 0.36 |
| 32 | 28.57 | 2.01 | 0.31 | 0.06 | 0.00 | 0.87 |
| 33 | 26.81 | 2.74 | 0.36 | 0.07 | 0.47 | 0.69 |
| 34 | 69.66 | 3.56 | 0.61 | 0.09 | 1.33 | 0.07 |
| 35 | 24.58 | 2.24 | 0.24 | 0.07 | 0.02 | 0.46 |
| 36 | 34.65 | 2.41 | 0.40 | 0.06 | 1.27 | 0.07 |
| 44 | 104.39 | 8.88 | 1.42 | 0.18 | 2.34 | 0.20 |
| 45 | 78.28 | 4.59 | 1.19 | 0.11 | 2.18 | 0.10 |
| 46 | 71.55 | 4.53 | 0.15 | 0.10 | 0.85 | 0.07 |
| 49 | 112.62 | 10.79 | 0.37 | 0.17 | 1.49 | 0.20 |
| 50 | 110.27 | 9.10 | 0.12 | 0.20 | 1.05 | 0.12 |
| 51 | 125.73 | 6.66 | 0.00 | 0.03 | 0.52 | 0.09 |
| 52 | 139.88 | 5.55 | 0.00 | 0.03 | 0.53 | 0.08 |
| 53 | 104.71 | 8.59 | 1.16 | 0.19 | 1.68 | 0.15 |
| 55 | 59.27 | 3.57 | 0.03 | 0.07 | 0.73 | 0.08 |

**Table S4**. Results from RD fits of the β region combined for GB1_dia_ at 14.1 and 16.4 T.

| Residue | *k*_ex_ (10^3^ s-^1^) | Error |  |  |  |  |
| --- | --- | --- | --- | --- | --- | --- |
| β region | 14.37 | 0.59 |  |  |  |  |
|  | ϕ_ex_ (10^3^ rad^2^ s-^2^) | Error | *R*_1ρ,0_ (s^-1^) at 16.4 T | Error | *R*_1ρ,0_ (s^-1^) at 14.1 T | Error |
| 12 | 63.91 | 9.95 | 1.88 | 0.21 | 7.29 | 0.28 |
| 17 | 49.07 | 9.22 | 2.90 | 0.33 | 4.15 | 0.19 |
| 18 | 54.78 | 5.54 | 1.40 | 0.16 | 3.15 | 0.19 |
| 19 | 96.79 | 4.13 | 0.27 | 0.09 | 2.76 | 0.08 |
| 20 | 86.84 | 8.17 | 1.17 | 0.16 | 4.78 | 0.26 |
| 44 | 106.18 | 7.31 | 1.40 | 0.15 | 2.32 | 0.16 |
| 45 | 78.02 | 4.26 | 1.18 | 0.09 | 2.18 | 0.10 |
| 46 | 72.17 | 4.28 | 0.13 | 0.10 | 0.85 | 0.07 |
| 49 | 114.09 | 8.49 | 0.38 | 0.14 | 1.45 | 0.18 |
| 50 | 111.33 | 7.62 | 0.11 | 0.16 | 1.05 | 0.11 |
| 51 | 125.57 | 5.45 | 0.00 | 0.02 | 0.54 | 0.08 |
| 52 | 140.41 | 5.13 | 0.00 | 0.01 | 0.54 | 0.07 |
| 53 | 103.55 | 8.40 | 1.18 | 0.19 | 1.71 | 0.15 |
| 55 | 59.40 | 3.58 | 0.04 | 0.08 | 0.73 | 0.08 |

**Table S5**. Results from RD fits of the α region combined for GB1_dia_ at 14.1 and 16.4 T.

| Residue | *k*_ex_ (10^3^ s-^1^) | Error |  |  |  |  |
| --- | --- | --- | --- | --- | --- | --- |
| α region | 15.85 | 1.52 |  |  |  |  |
|  | ϕ_ex_ (10^3^ rad^2^ s-^2^) | Error | *R*_1ρ,0_ (s^-1^) at 16.4 T | Error | *R*_1ρ,0_ (s^-1^) at 14.1 T | Error |
| 24 | 55.29 | 4.74 | 0.21 | 0.13 | 1.68 | 0.09 |
| 26 | 67.13 | 5.75 | 0.26 | 0.11 | 1.73 | 0.12 |
| 28 | 39.63 | 3.28 | 0.06 | 0.07 | 0.00 | 1.17 |
| 29 | 35.80 | 3.56 | 0.12 | 0.08 | 0.04 | 4.02 |
| 32 | 29.41 | 2.16 | 0.28 | 0.06 | 0.05 | 2.80 |
| 33 | 27.21 | 2.34 | 0.35 | 0.06 | 0.08 | 2.86 |
| 34 | 69.88 | 3.14 | 0.60 | 0.08 | 1.29 | 0.07 |
| 35 | 25.30 | 2.00 | 0.21 | 0.06 | 0.03 | 2.32 |
| 36 | 35.39 | 1.77 | 0.37 | 0.05 | 1.23 | 0.06 |

**Table S6**. Results from RD fits of individual residues for GB1_pre_ at 14.1 and 16.4 T. The χ^2^ minimization failed for residues 24 and 26.

| Residue | *k*_ex_ (10^3^ s-^1^) | Error | ϕ_ex_ (10^3^ rad^2^ s-^2^) | Error | *R*_1ρ,0_ (s^-1^) at 16.4 T | Error | *R*_1ρ,0_ (s^-1^) at 14.1 T | Error |
| --- | --- | --- | --- | --- | --- | --- | --- | --- |
| 12 | 22.36 | 6.66 | 104.57 | 19.32 | 6.16 | 0.34 | 3.30 | 0.35 |
| 17 | 24.89 | 8.13 | 81.54 | 22.00 | 7.14 | 0.38 | 4.38 | 0.41 |
| 18 | 15.89 | 5.22 | 52.18 | 10.62 | 6.27 | 0.27 | 5.40 | 0.39 |
| 24 | 1.00 | 3.08 | 207.65 | 134.91 | 6.85 | 0.26 | 4.89 | 0.29 |
| 26 | 1.00 | 1.66 | 368.51 | 199.17 | 3.36 | 0.14 | 2.29 | 0.17 |
| 28 | 3.23 | 1.97 | 86.33 | 76.03 | 3.17 | 0.12 | 2.49 | 0.15 |
| 29 | 15.82 | 5.02 | 33.57 | 6.43 | 3.25 | 0.17 | 2.41 | 0.18 |
| 32 | 23.62 | 5.60 | 44.84 | 8.22 | 3.51 | 0.16 | 2.33 | 0.16 |
| 33 | 28.87 | 5.76 | 53.17 | 9.70 | 2.43 | 0.15 | 2.03 | 0.18 |
| 35 | 8.48 | 3.43 | 30.89 | 5.24 | 4.23 | 0.17 | 3.12 | 0.19 |
| 36 | 1.79 | 1.73 | 83.01 | 73.80 | 4.07 | 0.07 | 2.99 | 0.09 |
| 44 | 16.66 | 4.14 | 61.74 | 7.36 | 3.27 | 0.19 | 2.54 | 0.20 |
| 45 | 35.50 | 5.89 | 111.02 | 17.69 | 2.44 | 0.21 | 1.87 | 0.23 |
| 46 | 19.33 | 2.55 | 74.47 | 5.88 | 2.01 | 0.12 | 1.33 | 0.13 |
| 49 | 18.09 | 6.43 | 124.08 | 16.68 | 5.04 | 0.38 | 3.54 | 0.42 |
| 50 | 4.47 | 3.94 | 85.15 | 129.91 | 5.69 | 0.31 | 3.96 | 0.36 |
| 51 | 11.76 | 2.29 | 109.49 | 10.64 | 3.24 | 0.27 | 2.44 | 0.33 |
| 52 | 15.51 | 4.99 | 63.14 | 7.36 | 1.96 | 0.17 | 2.00 | 0.18 |
| 53 | 20.98 | 4.92 | 74.56 | 11.76 | 2.95 | 0.25 | 2.29 | 0.25 |

**Table S7**. Results from RD fits of all residues combined for GB1_pre_ at 14.1 and 16.4 T.

| Residue | *k*_ex_ (10^3^ s-^1^) | Error |  |  |  |  |
| --- | --- | --- | --- | --- | --- | --- |
| All | 15.48 | 2.12 |  |  |  |  |
|  | ϕ_ex_ (10^3^ rad^2^ s-^2^) | Error | *R*_1ρ,0_ (s^-1^) at 16.4 T | Error | *R*_1ρ,0_ (s^-1^) at 14.1 T | Error |
| 12 | 95.58 | 15.34 | 6.23 | 0.39 | 3.38 | 0.41 |
| 17 | 62.64 | 17.13 | 7.35 | 0.52 | 4.68 | 0.46 |
| 18 | 59.02 | 11.89 | 6.13 | 0.39 | 5.26 | 0.43 |
| 24 | 37.91 | 23.31 | 6.85 | 0.65 | 4.73 | 0.50 |
| 26 | 67.19 | 8.48 | 2.96 | 0.26 | 1.96 | 0.23 |
| 28 | 59.39 | 6.54 | 2.80 | 0.20 | 2.15 | 0.21 |
| 29 | 40.21 | 8.12 | 3.10 | 0.25 | 2.35 | 0.22 |
| 32 | 43.12 | 5.99 | 3.52 | 0.21 | 2.32 | 0.19 |
| 33 | 39.32 | 6.04 | 2.62 | 0.19 | 2.22 | 0.21 |
| 35 | 36.74 | 8.55 | 3.96 | 0.27 | 3.01 | 0.25 |
| 36 | 36.66 | 3.70 | 3.75 | 0.12 | 2.73 | 0.13 |
| 44 | 73.22 | 9.21 | 3.11 | 0.28 | 2.43 | 0.23 |
| 45 | 71.92 | 9.35 | 2.85 | 0.27 | 2.36 | 0.22 |
| 46 | 78.55 | 8.60 | 1.99 | 0.24 | 1.27 | 0.21 |
| 49 | 124.65 | 25.81 | 5.06 | 0.50 | 3.60 | 0.58 |
| 50 | 64.97 | 28.17 | 5.51 | 0.72 | 3.70 | 0.58 |
| 51 | 121.37 | 18.61 | 2.83 | 0.49 | 2.24 | 0.46 |
| 52 | 87.31 | 11.03 | 1.66 | 0.28 | 1.77 | 0.22 |
| 53 | 81.59 | 11.56 | 2.78 | 0.36 | 2.24 | 0.29 |

**Table S8**. Results from RD fits of the β region combined for GB1_pre_ at 14.1 and 16.4 T.

| Residue | *k*_ex_ (10^3^ s-^1^) | Error |  |  |  |  |
| --- | --- | --- | --- | --- | --- | --- |
| β region | 14.22 | 1.93 |  |  |  |  |
|  | ϕ_ex_ (10^3^ rad^2^ s-^2^) | Error | *R*_1ρ,0_ (s^-1^) at 16.4 T | Error | *R*_1ρ,0_ (s^-1^) at 14.1 T | Error |
| 12 | 95.01 | 13.68 | 6.20 | 0.35 | 3.48 | 0.37 |
| 17 | 53.90 | 16.27 | 7.53 | 0.47 | 4.75 | 0.47 |
| 18 | 50.21 | 11.00 | 6.35 | 0.34 | 5.64 | 0.40 |
| 44 | 75.41 | 8.44 | 2.94 | 0.23 | 2.37 | 0.23 |
| 45 | 66.10 | 7.88 | 2.98 | 0.20 | 2.44 | 0.21 |
| 46 | 72.63 | 5.61 | 2.16 | 0.16 | 1.43 | 0.14 |
| 49 | 105.92 | 23.39 | 5.46 | 0.50 | 3.78 | 0.53 |
| 50 | 79.55 | 24.22 | 5.58 | 0.60 | 3.41 | 0.44 |
| 51 | 114.40 | 15.64 | 3.13 | 0.40 | 2.09 | 0.43 |
| 52 | 85.80 | 8.50 | 1.62 | 0.21 | 1.90 | 0.21 |
| 53 | 86.91 | 11.74 | 2.78 | 0.33 | 1.48 | 0.26 |

**Table S9**. Results from RD fits of the α region combined for GB1_pre_ at 14.1 and 16.4 T.

| Residue | *k*_ex_ (10^3^ s-^1^) | Error |  |  |  |  |
| --- | --- | --- | --- | --- | --- | --- |
| α region | 6.45 | 1.66 |  |  |  |  |
|  | ϕ_ex_ (10^3^ rad^2^ s-^2^) | Error | *R*_1ρ,0_ (s^-1^) at 16.4 T | Error | *R*_1ρ,0_ (s^-1^) at 14.1 T | Error |
| 24 | 53.77 | 12.64 | 6.68 | 0.28 | 4.75 | 0.28 |
| 26 | 79.26 | 12.65 | 3.25 | 0.16 | 2.20 | 0.18 |
| 28 | 61.79 | 7.32 | 3.06 | 0.15 | 2.39 | 0.16 |
| 29 | 35.49 | 5.38 | 3.38 | 0.13 | 2.53 | 0.17 |
| 32 | 37.12 | 4.27 | 3.82 | 0.12 | 2.61 | 0.13 |
| 33 | 30.79 | 2.89 | 2.92 | 0.11 | 2.50 | 0.14 |
| 35 | 34.16 | 5.59 | 4.25 | 0.15 | 3.14 | 0.17 |
| 36 | 38.63 | 4.44 | 3.95 | 0.08 | 2.88 | 0.10 |

**Table S10.** Statistical analysis of RD fits for all samples. Lowest BIC indicates that statistically in all cases the global fits are the best models. However, small difference in BIC suggest that the statistical significance is weak.

| Sample | Σ χ^2^ | Data points | Parameters | BIC |
| --- | --- | --- | --- | --- |
| **GB1_dia_** |  |  |  |  |
| All | 1327 | 460 | 70 | 1757 |
| α / β | 1326 | 460 | 71 | 1761 |
| **GB1_pre_** |  |  |  |  |
| All | 735 | 532 | 58 | 1099 |
| α / β | 753 | 532 | 59 | 1124 |
| **GB1:IgG** |  |  |  |  |
| All | 125 | 200 | 31 | 273 |
| Fab / Fc | 125 | 200 | 32 | 295 |

**Table S11**. Number of spin-lock lengths, number of nutation frequencies, recycle delay and total duration of each experiment.

| Sample | Spin-lock lengths | Nutation frequencies | Recycle delay | Total duration |
| --- | --- | --- | --- | --- |
| GB1_dia_ at 16.4 T | 10 | 13 | 2 s | 69.5 h |
| GB1_pre_ at 14.1 T | 8 | 11 | 0.2 s | 24 h |
| GB1_pre_ at 16.4 T | 10 | 17 | 0.8 s | 43 h |
| GB1:IgG at 16.4 T | 7 | 10 | 0.6 s | 231.5 h |
| GB1IgG at 20 T | 8 | 10 | 0.4 s | 141 h |

**Table S12**. Results from RD fits of individual residues for GB1 in complex with IgG at 16.4 and 20 T. The χ^2^ minimization failed for residues 11 and 19.

| Residue | *k*_ex_ (10^3^ s-^1^) | Error | ϕ_ex_ (10^3^ rad^2^ s-^2^) | Error | *R*_1ρ,0_ (s^-1^) at 20 T | Error | *R*_1ρ,0_ (s^-1^) at 16.4 T | Error |
| --- | --- | --- | --- | --- | --- | --- | --- | --- |
| 11 | 1.00 | 15.12 | 2544.45 | 2270.35 | 8.43 | 2.59 | 8.34 | 2.66 |
| 17 | 15.82 | 7.73 | 343.02 | 80.01 | 6.89 | 1.52 | 4.76 | 1.79 |
| 18 | 22.85 | 20.52 | 133.79 | 133.12 | 9.28 | 1.89 | 6.51 | 1.53 |
| 19 | 1.00 | 6.29 | 1047.21 | 825.72 | 9.66 | 0.80 | 5.72 | 0.55 |
| 20 | 32.23 | 14.49 | 311.30 | 147.23 | 11.63 | 2.34 | 6.18 | 1.94 |
| 38 | 16.64 | 13.81 | 177.23 | 206.68 | 11.32 | 1.71 | 9.27 | 1.58 |
| 44 | 16.26 | 10.37 | 167.83 | 106.71 | 8.56 | 1.14 | 5.85 | 1.15 |
| 49 | 19.19 | 19.31 | 245.98 | 512.36 | 11.49 | 2.58 | 7.15 | 2.22 |
| 50 | 24.51 | 31.12 | 120.58 | 219.09 | 9.47 | 2.51 | 7.58 | 1.91 |
| 51 | 8.83 | 5.28 | 303.65 | 378.72 | 10.64 | 0.92 | 5.36 | 0.81 |

**Table S13**. Results from RD fits of all residues combined for GB1 in complex with IgG at 16.4 and 20 T.

| Residue | *k*_ex_ (10^3^ s-^1^) | Error |  |  |  |  |
| --- | --- | --- | --- | --- | --- | --- |
| All | 13.17 | 2.53 |  |  |  |  |
|  | ϕ_ex_ (10^3^ rad^2^ s-^2^) | Error | *R*_1ρ,0_ (s^-1^) at 20 T | Error | *R*_1ρ,0_ (s^-1^) at 16.4 T | Error |
| 11 | 297.26 | 124.61 | 7.82 | 2.37 | 8.17 | 2.58 |
| 17 | 337.93 | 59.63 | 7.24 | 1.32 | 5.22 | 1.43 |
| 18 | 107.66 | 34.16 | 10.05 | 1.06 | 7.13 | 0.95 |
| 19 | 150.54 | 26.45 | 9.03 | 0.86 | 5.21 | 0.64 |
| 20 | 199.49 | 41.70 | 13.94 | 1.38 | 8.15 | 1.09 |
| 38 | 169.70 | 41.90 | 11.60 | 1.28 | 9.61 | 1.03 |
| 44 | 162.29 | 31.29 | 8.80 | 0.96 | 6.13 | 0.81 |
| 49 | 238.76 | 72.08 | 12.09 | 1.77 | 7.68 | 1.53 |
| 50 | 91.64 | 41.68 | 10.23 | 1.27 | 8.22 | 1.16 |
| 51 | 273.59 | 34.74 | 10.22 | 0.95 | 4.90 | 0.76 |

**Table S14**. Results from RD fits of residues expected to bind the Fab fragment of IgG combined for GB1 in complex with IgG at 16.4 and 20 T.

| Residue | *k*_ex_ (10^3^ s-^1^) | Error |  |  |  |  |
| --- | --- | --- | --- | --- | --- | --- |
| Fab region | 14.29 | 4.22 |  |  |  |  |
|  | ϕ_ex_ (10^3^ rad^2^ s-^2^) | Error | *R*_1ρ,0_ (s^-1^) at 20 T | Error | *R*_1ρ,0_ (s^-1^) at 16.4 T | Error |
| 11 | 290.75 | 118.32 | 7.73 | 2.42 | 8.13 | 2.61 |
| 17 | 339.24 | 60.84 | 7.09 | 1.41 | 5.01 | 1.55 |
| 18 | 109.42 | 36.85 | 9.95 | 1.11 | 7.05 | 0.94 |
| 19 | 149.44 | 27.91 | 8.97 | 0.85 | 5.16 | 0.67 |
| 20 | 202.12 | 38.30 | 13.79 | 1.22 | 8.00 | 1.02 |
| 38 | 171.66 | 42.11 | 11.51 | 1.30 | 9.49 | 1.13 |

**Table S15**. Results from RD fits of residues expected to bind the Fc fragment of IgG combined for GB1 in complex with IgG at 16.4 and 20 T.

| Residue | *k*_ex_ (10^3^ s-^1^) | Error |  |  |  |  |
| --- | --- | --- | --- | --- | --- | --- |
| Fc region | 12.27 | 2.90 |  |  |  |  |
|  | ϕ_ex_ (10^3^ rad^2^ s-^2^) | Error | *R*_1ρ,0_ (s^-1^) at 20 T | Error | *R*_1ρ,0_ (s^-1^) at 16.4 T | Error |
| 44 | 161.95 | 32.31 | 8.87 | 0.88 | 6.21 | 0.85 |
| 49 | 243.21 | 75.28 | 12.17 | 1.61 | 7.72 | 1.41 |
| 50 | 91.26 | 40.96 | 10.27 | 1.28 | 8.26 | 1.11 |
| 51 | 276.63 | 38.22 | 10.31 | 0.89 | 4.99 | 0.82 |

**References**

1 J. M. Lamley, C. Öster, R. A. Stevens and J. R. Lewandowski, *Angew. Chemie Int. Ed.*, 2015, **54**, 15374–15378.

2 H. L. F. Schmidt, L. J. Sperling, Y. G. Gao, B. J. Wylie, J. M. Boettcher, S. R. Wilson and C. M. Rienstra, *J. Phys. Chem. B*, 2007, **111**, 14362–9.
